# Micromachined Accelerometers with Sub-µg/√Hz Noise Floor: A Review

**DOI:** 10.3390/s20144054

**Published:** 2020-07-21

**Authors:** Chen Wang, Fang Chen, Yuan Wang, Sina Sadeghpour, Chenxi Wang, Mathieu Baijot, Rui Esteves, Chun Zhao, Jian Bai, Huafeng Liu, Michael Kraft

**Affiliations:** 1 College of Optical Science and Engineering, Zhejiang University, Hangzhou 310027, China; chen.wang@zju.edu.cn; 2 Department of Electrical Engineering and Computer Science, University of Liege, 4000 Liege, Belgium; 3 ESAT-MICAS, University of Leuven, 3001 Leuven, Belgium; sina.sadeghpourshamsabadi@kuleuven.be (S.S.); chenxi.wang@kuleuven.be (C.W.); mathieu.baijot@kuleuven.be (M.B.); ra.esteves@campus.fct.unl.pt (R.E.); 4 SIMIT, Chinese Academy of Sciences, Shanghai 200050, China; fangchen@mail.sim.ac.cn; 5 PGMF and School of Physics, Huazhong University of Science and Technology, Wuhan 430074, China; yuan.wang@uliege.be (Y.W.); chun_zhao@hust.edu.cn (C.Z.)

**Keywords:** micromachined/micro accelerometers, accelerometer structure, low-g accelerometer, seismometer, Micro-Electro–Mechanical System (MEMS), inertial sensors, accelerometer review

## Abstract

This paper reviews the research and development of micromachined accelerometers with a noise floor lower than 1 µg/√Hz. Firstly, the basic working principle of micromachined accelerometers is introduced. Then, different methods of reducing the noise floor of micromachined accelerometers are analyzed. Different types of micromachined accelerometers with a noise floor below 1 µg/√Hz are discussed. Such sensors can mainly be categorized into: (i) micromachined accelerometers with a low spring constant; (ii) with a large proof mass; (iii) with a high quality factor; (iv) with a low noise interface circuit; (v) with sensing schemes leading to a high scale factor. Finally, the characteristics of various micromachined accelerometers and their trends are discussed and investigated.

## 1. Introduction

High-resolution accelerometers are required for many specific application areas, including inertial navigation, earthquake detection, spacecraft guidance or stabilization, geophysical sensing, etc. The history of high-resolution accelerometers dates back to the 1960s, when they were used in space environment exploration missions. In the early 1970s, the Office National d’Etudes et de Recherches Aérospatiales (ONERA, France) developed an electrostatic accelerometer termed Capteur Accelerometrique Triaxial Ultra Sensible (CACTUS), designed for testing satellites, reaching a resolution of 10^−9^ m/s^2^ [1]. Shortly after, several similar high-resolution sensors were developed, such as the Super STAR and GRADIO accelerometers, which were employed for different satellites to conduct earth field recovery missions [2]. These early high-resolution accelerometers were macroscopic devices of large size and heavy weight, as well as high cost, and were fabricated by conventional macro-machining methods.

Compared to conventional macroscopic high-resolution accelerometers, micromachined accelerometers feature many benefits, such as small size, light weight, low cost, low power and easy integration with semiconductor technology. According to Middelhoek [3], the first Micro-Electro–Mechanical System (MEMS) accelerometer was developed by Vaganov in 1975, but he never published his work, as he was a visiting scientist from the USSR at Stanford University. Four years later, a piezoresistive MEMS accelerometer was developed by Roylance and Angell [4] (1979), reaching a resolution of a few mg and a bandwidth higher than 1 kHz. In the 1990s, driven by the development of automotive airbags, MEMS accelerometers took a huge step in their development, as they met the requirements for airbag systems (i.e., low cost, miniature in size, good reliability and the ability to detect the sharp acceleration shock generated by a car crash). In 1990, a MEMS accelerometer was put into an automobile for the first time and, in 2007, over 100 million MEMS accelerometers were sold that year [5]. More recently, smartphones have opened a considerable new market for MEMS accelerometers, as every smartphone produced nowadays contains a three-axis MEMS accelerometer [6]. These MEMS accelerometers have relatively low performance characteristics compared to high performance sensors used for the aforementioned applications; this is illustrated in Table 1 [5].

Various micromachined accelerometers have been reported in the last two decades. In 1998, a detailed review of micromachined inertial sensors was reported in which different categories of micromachined accelerometers and their key technologies were discussed [7]. However, the mechanical designs and the associated interface circuits of micromachined accelerometers have been evolving rapidly in recent years. In 2015, a review of high-g micromachined accelerometers was presented in [8]. However, low-g micromachined accelerometers (accelerometers with a sub-µg resolution are not discussed in [8].

In this review, we mainly focus on recent developments in micromachined structures and the circuit technologies of accelerometers with a sub-µg/√Hz noise floor. According to the methods of reducing the noise floor, these sensors can be categorized into micromachined accelerometers with: (i) a low spring constant; (ii) a large proof mass; (iii) a high quality factor; (iv) a low noise interface circuit; (v) other sensing schemes to achieve a high scale factor and low noise floor.

## 2. Micromachined Accelerometer Principles

**Figure 1:**
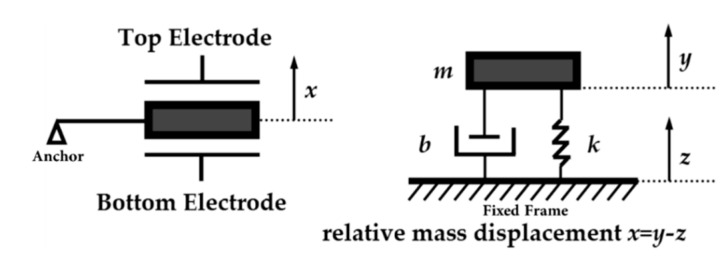
Schematic diagrams of a capacitive accelerometer structure and its mechanical lumped parameter model.

A micromachined accelerometer can be modeled to first-order approximation by a spring–mass–damp system, as shown in Figure 1. It consists of a proof mass *m* suspended by a compliant suspension system with an effective spring constant *k*. A viscous damping factor *b* is introduced to describe the effect of damping on the dynamic characteristic of the accelerometer, as squeeze and slide film damping are important effects due to the small size of the micromachined sensing element. The proof mass moves relative to a fixed frame under an acceleration input. Different transduction methods can be used to measure the displacement of the proof mass and hence determine the acceleration input. The transfer function of the motion of the micromachined accelerometer is given by Equation (2), where *ω_0_* is the mechanical resonance angular frequency, *m* is the proof mass weight, *k* is the spring constant, *b* is the damping factor and *Q* is the quality factor.

(1) $$Q=\sqrt{\frac{km}{b}}$$

(2) $$H(s)=\frac{x(s)}{a(s)}={\left(s^{2}+\frac{bs}{m}+\frac{k}{m}\right)}^{-1}={\left(s^{2}+\frac{\omega_{0}}{Q}+\omega_{0}{}^{2}\right)}^{-1}$$

The resolution of a MEMS accelerometer is determined by calculating the total noise equivalent acceleration (*NEA*), which consists of the circuit noise equivalent acceleration (*ENEA*), the Brownian noise equivalent acceleration (*TNEA*) as well as the quantization noise equivalent acceleration (*QNEA*), as the output is usually digitized. *NEA* is influenced by both the sensitivity of the accelerometer and the input referred noise of the sensor system.

(3) $$NEA=\sqrt{TNEA^{2}+ENEA^{2}+QNEA^{2}}$$

*TNEA* primarily originates from the Brownian motion of air molecules around the micromachined proof mass and can be expressed by,

(4) $$TNEA=\sqrt{\frac{4k_{B}Tb}{m}}=\sqrt{\frac{4k_{B}T\omega_{0}}{mQ}}$$

where *T* is the ambient temperature and *k_B_* is the Boltzmann constant. *TNEA* can be lowered by improving the weight of the proof mass, reducing the resonant frequency and improving the quality factor (*Q*). If the proof mass weight and the suspension spring constant of a MEMS accelerometer remains unchanged, *Q* is manly influenced by air damping. However, accelerometers with high *Q* require a closed-loop control system to ensure stability, as the sensor could easily exhibit unstable behavior, such as a long settling time and a large overshoot that can lead to saturation both in the mechanical as well the electrical domain. To some extent, this compromises the benefits of low *TNEA* due to a high *Q* [9,10].

MEMS accelerometers do not measure the acceleration signal directly but through the measurement of the displacement of the proof mass or the mechanical stress on the suspension system, resulting from the acceleration inertial force. This can be achieved by various transduction methods, such as capacitive, piezoresistive, piezoelectric, thermal, optical, electromagnetic, tunneling effect, etc. Therefore, *ENEA* is inversely proportional to the scale factor of the accelerometer and the interface circuit input referred noise. The total scale factor of the accelerometer can be increased by improving the mechanical scale factor (by lowering the spring constant and increasing the weight of the proof mass) or applying other design methods, resulting in a higher scale factor, such as mechanical displacement amplification.

Since most of the MEMS accelerometers, which will be discussed in the following sections, used capacitive sensing, we take MEMS accelerometers with a gap changing capacitive sensing method as an example to discuss *ENEA*. *ENEA* can be expressed by,

(5) $$ENEA=\frac{\Delta C_{\min}}{SF_{cap}}$$

(6) $$SF_{cap}\approx \left(\frac{m}{k}\right)\cdot \left(\frac{\varepsilon_{0}\varepsilon_{r}A}{d^{2}}\right)=\frac{\varepsilon_{0}\varepsilon_{r}Am}{kd^{2}}$$

where Δ*C_min_* is the input referred noise of a capacitive sensing circuit. *SF_cap_* is the capacitive sensing scale factor (ratio of capacitive change and acceleration input). *A* is the overlap area of capacitive comb fingers. *d* is the gap between the capacitive comb fingers. *ε_0_* is the permittivity of vacuum and *ε_r_* is the relative permittivity of air (assumed to be as one here).

The frequency sensing scheme is used as an example to discuss *ENEA*, which was discussed in Section 6.2.1. *ENEA* can be expressed by,

(7) $$ENEA=\frac{\Delta f_{\min}}{SF_{res}}$$

(8) $$SF_{res}\approx \frac{1}{4}mf_{c}S=\frac{0.293}{4}mf_{c}\left(\frac{L_{T}{}^{2}}{Et_{T}w_{T}{}^{3}}\right)$$

where Δ*f_min_* is the input referred noise of a frequency sensing circuit. *SF_res_* is the frequency sensing scale factor (ratio of frequency change and acceleration input). *E* is the modulus of elasticity of the material. *L_T_*, *w_T_* and *t_T_* are the length, width and structural thickness of the laterally vibrating beam attached to the proof mass, respectively. *f*_c_ is the resonant frequency of the laterally vibrating beam attached to the proof mass without axial force load.

*QNEA* mainly results from the quantization process in an analog to digital converter (ADC) or a digital to analog converter (DAC) in the signal chain of an accelerometer sensor system. ADC is typically used at the output of an open-loop sensor or in the feedforward loop of a digital closed-loop control system, such as an electromechanical sigma–delta modulator (ΣΔM) [11,12]. DAC are usually required in the force feedback loop of a digital closed-loop control system. For a N-bit quantizer, if the input signal remains within the quantizer full scale level range (*V_fs_*) and changes sufficiently fast, the quantization error can be treated as white noise, which is independent of the input signal and has equal probability to lie between −∆/2 and ∆/2 (∆ is the space between two adjacent quantization levels).

*QNEA* can be described by:

(9) $$QNEA=\frac{\frac{\Delta }{\sqrt{12}}}{SF\cdot EG}=\frac{V_{fs}}{2^{N}\sqrt{12}}\cdot \frac{1}{SF\cdot EG}$$

where *EG* refers to the electronic gain of the interface circuit (including charge amplifiers, instrumental amplifiers and boost amplifiers). *SF* is the scale factor of different sensing schemes (e.g., *SF_cap_*, *SF_res_*). The quantization noise in the feedforward loop can be reduced by increasing the number of bits. For closed-loop control systems, the quantization noise can be reduced by increasing the oversampling ratio or implementing a high order (order > 2) electromechanical ΣΔM, which can shape the quantization noise out of the frequency band of interest to improve the noise floor.

It is worth mentioning that to reduce the noise floor, most high-resolution accelerometers operate in an open-loop mode to exclude the introduction of additional electronic noise by the feedback electronics. High-resolution MEMS accelerometers are typically used for applications with a relatively small full scale range, making closed-loop systems obsolete [13]. Sensors with a high Q sensing element, with a requirement of large dynamic range or high bias stability, however, typically require closed-loop control.

A variety of methods have been investigated for micromachined accelerometers to achieve a noise floor below 1 µg/√Hz. In the following sections, different approaches are reviewed. They are classified into five main categories, which are accelerometers with: (i) a low spring constant; (ii) a large proof mass; (iii) a high Q; (iv) a low noise interface circuit; (v) high scale factor transduction schemes. Some micromachined accelerometers with a noise floor above 1 µg/√Hz, which have a potential to achieve a sub-µg/√Hz noise floor, are also discussed.

## 3. MEMS Accelerometers with Novel Mechanical Design

### 3.1. MEMS Accelerometers with a Low Spring Constant

#### 3.1.1. MEMS Accelerometers with Geometric Anti-Spring (GAS)

**Figure 2:**
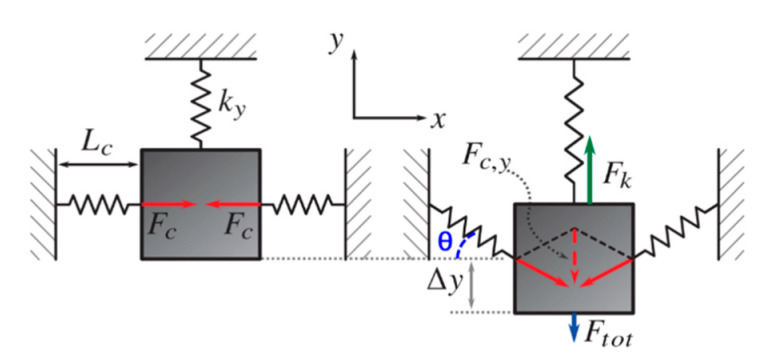
Conceptual illustrations of a MEMS accelerometer with GAS. Left: a proof mass in an equilibrium position is connected to a spring in the y-direction with stiffness *k_y_* and two springs forming the GAS in the x-direction of stiffness *k_c_*, producing a lateral preloading force *F_c_* as the springs with stiffness *k_c_* are compressed to a length *L_c_*_._ Right: the proof-mass in a displaced state with a displacement of Δ*y*, introducing a rotation *θ* and a restoring force F_k_ = −k_y_*Δ*y*. *F_c,y_* is the sum of the parts of *F_c_* along the axis of sensitivity. *F_tot_* is partly canceled by *F_c,y_* [13,16].

In gravitational wave detectors, GAS technology is used to reduce the resonance frequency of a large seismic isolation filter, which reduces the attenuation of the gravitational wave signal by external seismic activity by a factor of about 10^12^ [14,15]. A similar principle can be used for MEMS accelerometers, for which the GAS typically consists of a set of curved, pre-stressed springs attached to the proof mass. The springs can be compressed or loaded passively with a force, due to earth gravity, or actively with a dedicated actuation system. The compression of the springs effectively decreases the resonance frequency along the axis of the sensitivity of the mass-spring system, which significantly increases the sensitivity of the accelerometer. As shown in Figure 2, a proof mass is connected to a spring in the y-direction of stiffness *k_y_* and two springs forming the GAS in the x-direction of stiffness *k_c_*, which is pre-loaded with a force *F_c_* in the direction orthogonal to the sensing direction and compressed to a length *L_c_*. The system is in equilibrium since the compression forces *F_c_* cancel each other out.

If there is a displacement of the proof mass by Δ*y*, the horizontal spring rotates by a small angle *θ* and thus a force *F_c,y_* arises along the axis of sensitivity expressed by,

(10) $$F_{c,y}=2F_{c}\sin\theta$$

Assuming *θ* as small,

(11) $$\sin\theta \approx \tan\theta =\frac{\Delta y}{L_{c}}\approx \theta$$

Thus, the relation between proof mass displacement Δ*y* and *F_c,y_* is,

(12) $$F_{c,y}=2F_{c}\frac{\Delta y}{L_{c}}$$

The force equilibrium in the vertical direction is described by Equation (12). *F_c,y_* cancels parts of the restoring force F_k_ = −k_y_* Δ*y*, generated by the suspension springs, and effectively lowers the total restoring force *F_tot_* to,

(13) $$F_{tot}=F_{k}-F_{c,y}=-\left(k_{y}-2\frac{F_{c}}{L_{c}}\right)\Delta y$$

The total spring constant *k_tot_* in the vertical direction can then be expressed by,

(14) $$k_{tot}=\frac{F_{tot}}{\Delta y}=k_{y}-2\frac{F_{c}}{L_{c}}$$

indicating that the compression of the curved springs in the horizontal direction can reduce the total spring constant.

**Figure 3:**
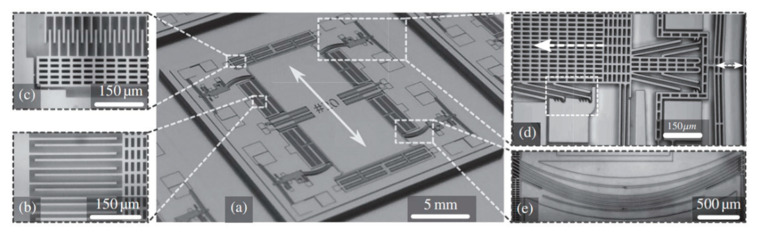
SEM images of MEMS accelerometer developed by Boom and Kamp et al. at Nikhef and University of Twente [13,16]: (**a**) entire sensor chip, (**b**) sense comb fingers, (**c**) actuation electrodes, (**d**) preloading mechanisms, (**e**) one of the suspension spring sets in a compressed state.

Boom and Kamp et al. [13,16] (2015) at Nikhef and the University of Twente developed an ultra-sensitive MEMS accelerometer with GAS technology with a noise floor below 2 ng/√Hz (at 28.1 Hz), as shown in Figure 3. An active preloading mechanism was utilized to bend the spring and, in this manner, the spring constant was reduced by a factor of 26 to a small positive value close to 0 N/m in the sense direction. Thus, the sensitivity to acceleration was also increased by the same factor. As shown in Figure 3d, in the preloading mechanism, by electrostatically compressing the V-beam on the right-hand side, the linear guiding block attached to the proof-mass on the left hand can be stepped forward to compress the spring with a displacement of over 35 μm. This preloading causes a stiffness reduction by a purely mechanical mechanism, which is independent of the proof-mass position and thus maintains good linearity. Moreover, no power was consumed after the accelerometer was put in its preloaded state.

**Figure 4:**
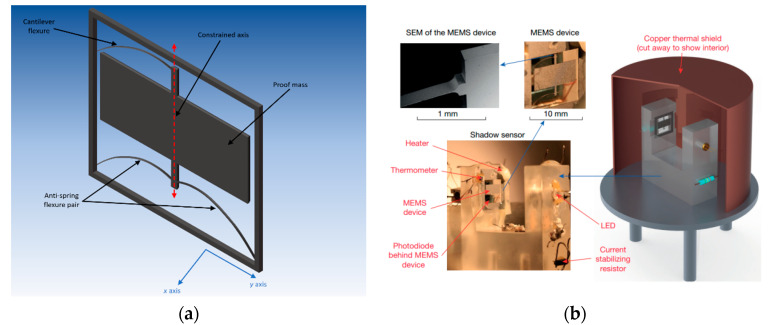
Schematic diagrams of MEMS accelerometer and experimental setup developed by Middlemiss et al. at University of Glasgow [17]: (**a**) schematic diagram of the MEMS accelerometer—central proof mass was suspended from three flexures: an anti-spring pair at the bottom and a curved cantilever at the top; (**b**) experimental setup. (Figure 4a is redrew by the authors)

Middlemiss et al. [17] (2016) at the University of Glasgow applied the GAS approach to a MEMS accelerometer design to reduce the stiffness of the suspension, which is illustrated in Figure 4. The sensor achieved a noise floor of 40 ng/√Hz and a low resonant frequency of 2.3 Hz. This novel design comprised three springs, in which the nonlinearity of the two GAS was combined and further increased the dynamic range of the device. The spring was passively preloaded by the force of gravity. An optical sensor was used to measure the displacement of the proof mass in which a light-emitting diode (LED) illuminated the MEMS structure, placed between the light source and an optical detector. Under acceleration, the displacement of the proof mass modulated the light intensity on the optical detector and thus the output current of a photodiode. This optical detector achieved an acceleration equivalent noise floor of ≤10 ng/√Hz at a sampling frequency of 0.03 Hz and a large dynamic range of up to 0.344 mg. Prasad et al. [18] (2018) at the University of Glasgow further presented a field-portable MEMS accelerometer with a noise floor of 8 ng/√Hz (at 1 Hz), which represented a 4–5 times improvement compared to earlier versions [17]. The sensor was packaged in a 10 cm × 10 cm vacuum enclosure, comprising the MEMS sensor, heater, thermometer, optical sensor as well as an adjustable tilt. The temperature effect was suppressed by a fused silica and a copper thermal shield. Several measurement results with the integrated platform have been reported by Middlemiss et al. [19,20]. Thanks to its long-term stability and low noise floor, the accelerometer was the first MEMS accelerometer that could measure the earth tides.

However, the optical shadow sensor in [17] is bulky and expensive, which prevents the miniaturization and wide use of such accelerometers. EI Mansouri et al. [21] (2019) at Delft University of Technology developed a MEMS accelerometer with GAS comprising an application-specific integrated circuit (ASIC) readout, based on the design in [17]. The accelerometer has a theoretical noise floor of 17.02 ng/√Hz and a resonant frequency of 8.7 Hz. It is designed with a capacitive readout scheme (integrated on the same bulk micromachined silicon die) through a trench isolation technique, which electrically separates both parts of the silicon bulk while keeping the lithographic lateral resolution. The presented concept shows the potential for an integrated platform of an extremely compact MEMS + ASIC gravimeter, both at the system-in-a-package level, or even a monolithically integrated CMOS on the same die as the MEMS device.

Zhang et al. [22] (2019) at Xi’an Jiaotong University and Changan University developed a MEMS accelerometer with anti-spring structures with a noise floor of 52 ng/√Hz (at 1 Hz) within a bandwidth of 158 Hz. The anti-spring structures were pre-loaded through electrostatic force produced by capacitive comb fingers.

**Figure 5:**
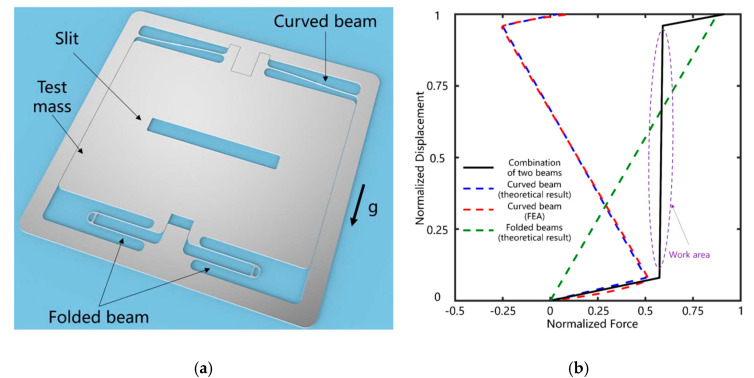
Schematic diagram of the MEMS accelerometer with a novel quasi-zero stiffness suspension developed by Tang et al. at Huazhong University of Science and Technology and its spring constant [23]: (**a**) schematic diagram of the MEMS mechanism; (**b**) normalized force-displacement curve of different spring designs.

Tang et al. [23,24] (2019) at Huazhong University of Science and Technology developed a MEMS accelerometer with a novel quasi-zero stiffness suspension, as shown in Figure 5. In this design, the negative spring constant of a curved bi-stable spring cancelled the positive spring constant of two folded springs, producing a low overall spring constant and thus a low resonant frequency of 3 Hz. It successfully measured the earth tides with a noise floor of 8 ng/√Hz (at 1 Hz), a dynamic range of 8 mg and a bandwidth from 0.5 Hz to 3 Hz.

The negative spring constant of GAS can reduce the spring constant of a MEMS accelerometer to nearly 0 N/m and hence improve its sensitivity. Besides, such MEMS accelerometers exhibit excellent long-term stability, and thus can measure signals with a frequency as low as 1 × 10^−5^ Hz. However, the low resonant frequency resulting from the low spring constant limits the bandwidth and the dynamic range of such accelerometers.

#### 3.1.2. MEMS Accelerometers with Materials of Low Young’s Modulus

**Figure 6:**
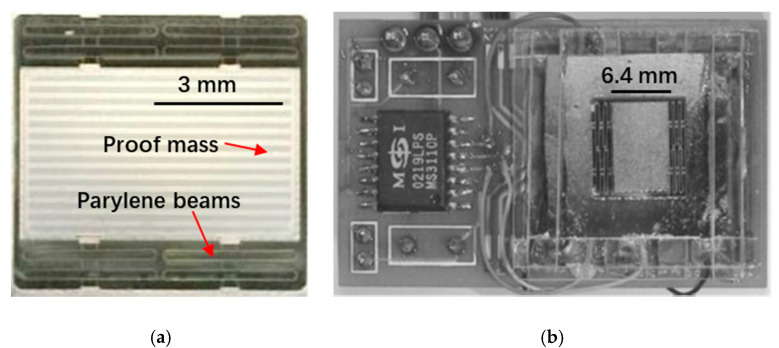
Images of the parylene based capacitive accelerometer developed by Suzuki et al. at University of Tokyo and California Institute of Technology [25]: (**a**) top view of the accelerometer; (**b**) image of the interface of the circuit and the accelerometer.

The spring constant of a suspension system is not only influenced by the geometry of the springs but also the Young’s modulus of the material used. Thus, by using a material with low Young’s modulus, more compliant springs can be used to achieve higher sensitivity. Suzuki et al. [25] (2003) at the University of Tokyo and the California Institute of Technology developed a suspension system made of parylene to reduce the spring stiffness of MEMS accelerometers. Parylene has a smaller Young’s modulus compared to silicon and is non-brittle, as shown in Figure 6. The parylene beams with a width of 10–40 μm and an aspect ratio between 10 and 30 were successfully fabricated, achieving a spring constant in the order of 1 × 10^−3^ N/m. A capacitive accelerometer was made with a noise floor of 45 µg/√Hz and a resonant frequency of 37 Hz. If the sensing element was designed with a higher scale factor or an interface circuit with a lower noise floor (an interface circuit with 4 aF/√Hz was used in this work, but Si-WARE SWS1120, for example, has a noise floor of 50 zF/√Hz [26]) was used, such a sensor could achieve sub-µg resolution as the *TNEA* of the device was only 25 ng/√Hz.

It is worth mentioning that polymer is inferior to silicon in terms of the long term stability of its mechanical properties and thermal expansion coefficient, which make polymer less suitable for the implementation of low noise MEMS accelerometers.

### 3.2. MEMS Accelerometers with a Large Proof Mass

As mentioned in Section 2, a large proof mass can result in low *TNEA* and a high mechanical scale factor. A large proof mass can be achieved by increasing its area or thickness (usually fabricated through bulk micromachining using one or several deep reactive ion etching (DRIE) process steps) and using a material with high density.

**Figure 7:**
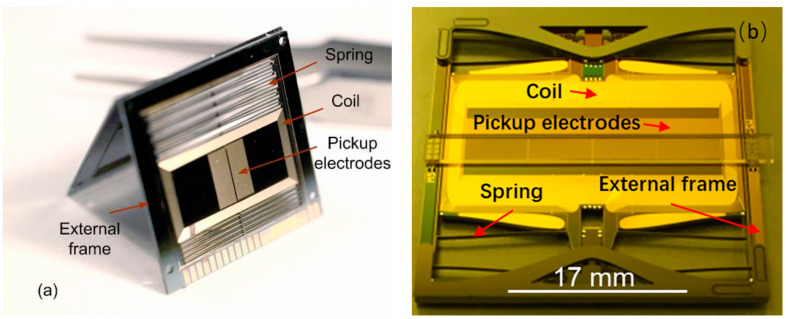
Photographs of the first version of MEMS accelerometers developed by Pike et al. at Imperial College: (**a**) first-generation MEMS accelerometer [27], (**b**) latest version [32].

Pike et al. at Imperial College developed various MEMS accelerometers with large proof masses for seismic monitoring in both terrestrial and planetary deployment. A “sandwich” area changing capacitive MEMS accelerometer with a proof mass of 0.4 g in a die size of 21 mm^2^ was reported by Pike and Kumar et al. [27] (2009), with a noise floor of 4 ng/√Hz (at 0.1 Hz) operating in a closed-loop working mode, as shown in Figure 7a. Such a low noise floor in a MEMS accelerometer was achieved by maximizing the proof mass with through-wafer etching. Weak flexures, oriented in the <100> direction of single-crystal silicon, were used to achieve a low resonant frequency. Gas damping was minimized by the suspension, transduction and packaging design and geometry, resulting in a relatively high Q of 252. On a single crystal silicon wafer, functional structures, including the proof mass, springs, feedback coils and capacitive electrodes, were fabricated and two glass wafers with cavities were used as upper and lower covers, respectively. The etch process parameters were optimized to improve the sidewall smoothness, and the detrimental artifacts of the DRIE plasma processing were eliminated to improve fracture strength. An intermediate frame was combined with conventionally folded springs to suppress out-of-plane motion and increase the frequencies of spurious off-axis modes [28]. A feedback system, based on electromagnetic actuators, was built for the accelerometers. Two sets of external magnets of flattened horseshoe geometry were put on each side of the assembled dies. A set of rectangular spiral coils, metalized on the proof mass, was connected to the external electronics. As current flows through the coil, the proof mass experiences a lateral feedback force from both arms of the magnetic coil. In 2013, solder bumpers were added to protect the proof mass from impact shocks of up to 2000 g during landing and take-off.

Pike et al. [29] (2014) reported a further optimized version with a noise floor of 2 ng/√Hz (around 1 Hz) and a large operation range of deployment tilt (±20°). The new design optimized the trade-off between damping induced noise and the displacement gain of the capacitive transducer. The optimization was achieved by making the area with minimum gaps to achieve a maximum proof mass weight and by allowing enough gas flow through a thin strip between the proof mass and the glass covers. Liu and Pike [30] (2015), the same group, introduced a novel thermal compensation scheme to passively counteract the thermal drift of the suspension system subject to gravity with a thermal actuator using solder encapsulated by silicon cavities. The temperature coefficient of the sensitive axis of the accelerometer displacement was reduced from −60 ppm/K to less than 1 ppm/K. By reducing the power of each noise source and maximizing the amplitude of the previous gain stage in the signal pathway, Pike et al. [31,32,33] (2016) reported another optimized version, achieving a noise floor of 0.25 ng/√Hz in a frequency band from 0.1 to 10 Hz. It had high shock robustness (>1000 g) and could operate from +60 C to −80 C, as shown in Figure 7b. The weight of the proof mass was maximized, both by enlarging the die area and by the addition of gold bars, doubling the mass from 0.4 to 0.8 g. An electrode spacing of 12 µm and periodicity of 48 µm minimized noise injection from surface-state fluctuations and increased the displacement transducer (DT) gain. Noise injection from the front-end electronics were controlled by a suitable preamplifier, minimizing both voltage noise floor and parasitic capacitance that would otherwise attenuate the DT amplifier. Additionally, the DT was operated in a modulated mode, well above the corner frequency at which flicker noise rises above the thermodynamic noise level.

**Figure 8:**
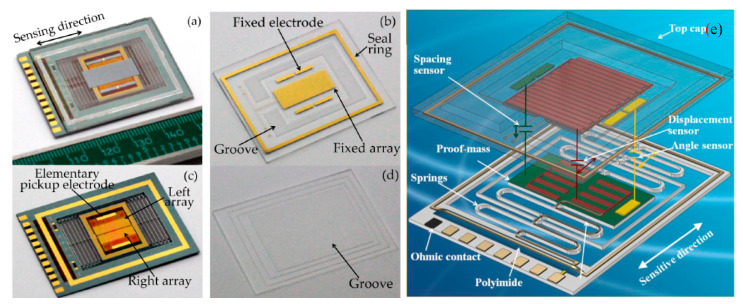
Images of the MEMS accelerometer developed by Wu et al. at Huazhong University of Science and Technology [39]: (**a**) assembled MEMS accelerometer; (**b**) top cap; (**c**) suspended proof-mass; (**d**) bottom cap; (**e**) schematic drawing of the MEMS accelerometer.

Wu and Li et al. [34,35,36,37,38,39] (2015) at Huazhong University of Science and Technology also developed similar accelerometers for gravity gradient measurements. They also relied on a large proof mass but had a higher noise floor and a larger dynamic range compared to the accelerometers from Imperial College [28]. As shown in Figure 8, the accelerometer used three displacement sensors (encoder-like area changing capacitive sensors) to achieve both a large dynamic range and a high sensitivity. The first two displacement sensors had a high sensitivity but low measuring range. They worked alternatively to avoid inflection points [40], which resulted from a sinusoidal periodic output signal of a general encoder-like area changing capacitive sensors. The phase of the sinusoidal periodic outputs of the two displacement sensors were shifted by a quarter of the period, so that when one array operated near the inflection points, the other one had to operate in its linear range. Thus, the seismic sensor could operate linearly with a high sensitivity, while being mounted at an arbitrary inclination angle. Furthermore, a third displacement sensor with a coarse sensitivity but a large measuring range was employed to select the working point array for the two displacement sensors. However, the sensitivity of the area changing capacitance was limited by parasitic capacitances and fringing field effects, which became significant when the electrode spacing of the capacitors was larger than the width of a single electrode. Wu, et al. [34,35,36,37] (2017) reduced the parasitic capacitance between the electrodes on the proof mass and the substrate from 1000 pF to 406 pF by inserting 3 μm photosensitive polyimide. The optimized accelerometer had a noise floor of 30 ng/√Hz (at 1 Hz), a resonant frequency of 13.2 Hz, as well as a full scale range of ±1.4 g [34]. Wu et al. [38] (2018) further reduced the electrode spacing from 90 μm to 20 μm using a three dimensional (3D) electroplating process. This improvement resulted in a noise floor of 10 ng/√Hz at 1 Hz [38]. Wu et al. [39] (2018) further integrated two other capacitive sensors into one chip (i.e., an angle sensor and a gap changing capacitive displacement sensor). An angle sensor (similar to the pick-up electrodes) selected the working array from the two arrays and measured the angle between the axis of sensitivity and the direction of gravity at the same time. In order to calibrate the scale factor, a gap-varying capacitive displacement sensor was integrated to measure the spacing. Through the combination of these capacitive sensors, the MEMS seismic sensor was able to work at arbitrary inclination angles with a resolution better than 50 ng/√Hz (at 1 Hz) [39].

**Figure 9:**
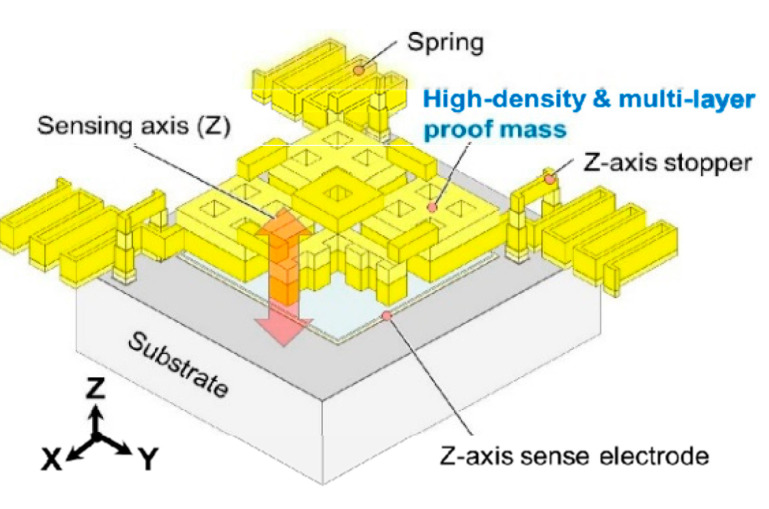
Schematic diagrams of the MEMS accelerometer developed by Yamane et al. at Tokyo Institute of Technology [42].

The proof mass weight can also be increased with a material of higher density, such as gold. The *TNEA* of a proof mass made of gold (density 19.3 × 10^3^ kg/m^3^ at 298 K [41]) is nearly one order larger than that made of silicon (2.33 × 10^3^ kg/m^3^ at 298 K [41]) for the same dimension. As shown in Figure 9, Yamane et al. [42] (2016) at the Tokyo Institute of Technology developed a MEMS accelerometer with a Brownian (thermal–mechanical) noise floor of 22 ng/√Hz, which consisted of a multi-layer metal proof mass structure made of electroplated gold. It was suggested that such an accelerometer could achieve a sub-µg resolution by using a higher resolution interface circuit, which is discussed in Section 5.

Edalatfar et al. at Simon Fraser University developed various MEMS accelerometers with high resolutions and large bandwidths. Edalatfar et al. [43] (2016) developed a capacitive MEMS accelerometer with a resonant frequency of 2 kHz and a thermal noise floor of 193 ng/√Hz. In 2018, a capacitive MEMS accelerometer for the detection of sonar waves was developed based on a mode-tuning structural platform, which had a noise floor of lower than 350 ng/√Hz (at 1–5 kHz) and a dynamic range of 135 dB [44]. To achieve a high sensitivity, the accelerometers were designed with a large number of capacitive combs with small gaps (2.2 μm) and a large proof masse. However, such design necessitated a large area, leading to undesired out-of-plane vibration modes (including some asymmetric modes) that were introduced into the bandwidth of the device. Subsequently, a frame was used instead of a solid plate for the proof mass of the device, which pushed undesired vibration modes beyond the operating bandwidth by carefully adding anchors and elastic frames on both the inside and outside of the moving frame.

Yazdi at Arizona State University and Najafi at the University of Michigan [45] (2000) developed a MEMS accelerometer that was fabricated on a single silicon wafer with a combined surface and bulk fabrication process. Accelerometers with 2 mm × 1 mm and a 4 mm × 1 mm proof masses were fabricated and had a noise floor at atmospheric pressures of 230 ng/√Hz and 160 ng/√Hz, respectively. The sense/feedback electrodes were formed by the deposition of 2–3 µm polysilicon film with embedded 25–35 µm-thick vertical stiffeners. These electrodes, while thin, were stiffened by the thick embedded structures so that the force rebalancing of the proof mass became possible. The polysilicon electrodes were patterned to create damping holes. The accelerometer reached a bandwidth higher than 1 kHz.

**Figure 10:**
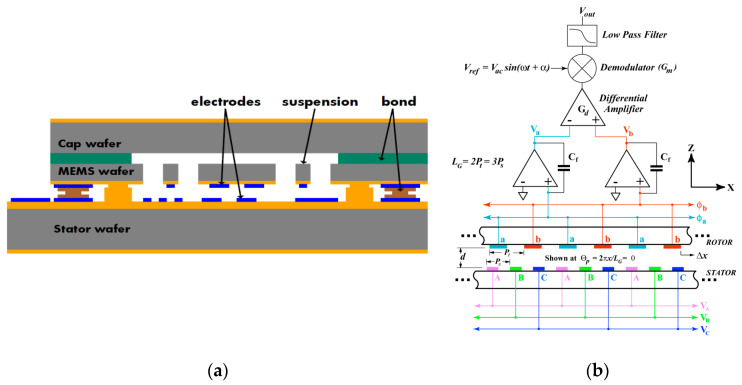
Schematic diagrams of the Hewlett Packard MEMS accelerometer and its sensing scheme: (**a**) schematic cross-section of the MEMS accelerometer [46]; (**b**) HP’s three phase capacitive sensing electrode arrangement.

The company Hewlett Packard (USA) [46] (2010) developed an open-loop MEMS accelerometer with a large proof mass (around 5 × 5 mm^2^) aimed for seismic imaging applications, as shown in Figure 10. The sensor had a noise floor lower than 10 ng/√Hz (at 1–200 Hz) and a dynamic range of 120 dB. With a custom designed ASIC interface, the sensor had a noise floor lower than 10 ng/√Hz down to 1 Hz. Such high sensitivity was achieved through a large capacitive sensing area and a small electrode gap. The sensing scheme, called three-phase sensing, enabled a purely electrical method for the compensation of fabrication tolerances [47], which trimmed the sensor output for canceling bias signal or nulling offsets, allowing the sensor to be operated in a regime of maximum sensitivity and linearity, regardless of overall orientation, fabrication errors, or other effects.

Many other companies also developed different capacitive MEMS accelerometers with a large proof mass, as listed in Table 2. The company Safran Colibrys (Yverdon-les-Bains, Switzerland) developed different types of capacitive MEMS accelerometers, such as SF1500 (300 ng/√Hz (at 10–1000 Hz)), SF2005S.A (800 ng/√Hz (at 10–1000 Hz)), SF3000 (300 ng/√Hz (at 10–1000 Hz)), SI1000 (700 ng/√Hz (at 0.1–100 Hz)), which all are vacuum packaged [48]. The company Kinemetrics (Pasadena, CA, USA) developed two types of capacitive MEMS accelerometers; i.e., EpiSensor ES-T (60 ng/√Hz), EpiSensor ES-U2 (60 ng/√Hz), EpiSensor 2 (3 ng/√Hz (at 1 Hz)), which are available in single and three-axis configurations [49]. The company Reftek (Nova Scotia, NS, Canada) developed two types of capacitive MEMS accelerometers; i.e., 131A (14 ng/√Hz) and Trimble 147A (10 ng/√Hz) [50]. The company Sercel (Nantes, France) developed a capacitive MEMS accelerometer with a proof mass size of 5.3 × 2.6 mm^2^, operating in closed-loop mode (DSU1-508) with a noise floor of 15 ng/√Hz (at 10–200 Hz) and DSU-3 with a noise floor of 41 ng/√Hz (at 10–200 Hz) [51,52]. The company INOVA (formerly Input/Output, Falls Church, VA, USA) developed two types of capacitive MEMS accelerometers, ACCUSEIS SL11 (30 ng/√Hz (at 3–400 Hz)) and VECTORSEIS ML21 (40 ng/√Hz (at 3–375 Hz)) [53].

A large proof mass makes a MEMS accelerometer experience a large inertial force under an acceleration input. Such types of MEMS accelerometers have recently demonstrated groundbreaking resolution and noise floor. However, the physics of the approach limits its scalability due to the direct relationship between the mass of the accelerometer and its sensitivity, and the inverse-squared relationship with the mechanical bandwidth of the accelerometer. These trends partially offset the benefits offered by miniaturization and can limit inherent device robustness.

## 4. MEMS Accelerometers with Vacuum Packaging

Since *TNEA* is inversely proportional to Q, the inherent noise floor of the accelerometer can be reduced by improving Q through vacuum packaging if the proof mass weight suspension spring constant of a MEMS accelerometer remains unchanged. However, high Q results in sensors with long settling time and large overshoot, thus require a closed-loop system to maintain a stable measurement, which may, to some extent, compromise the benefit of low *TNEA* due to high Q.

**Figure 11:**
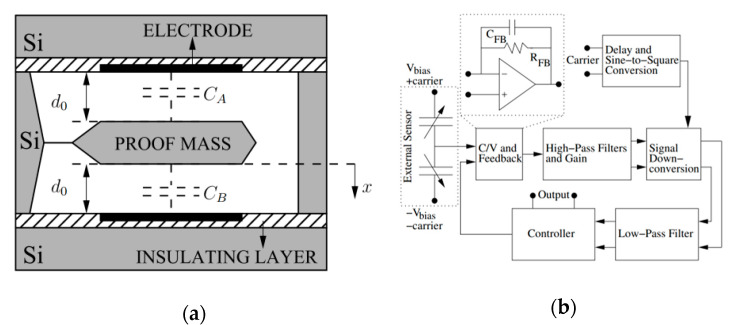
Schematic diagrams of the MEMS accelerometer and its closed-loop system developed by Aaltonen et al. at Helsinki University of Technology [9,10]: (**a**) schematic of the MEMS accelerometer; (**b**) block diagram of the closed-loop control system.

Wu et al. [12] (2006) at Carnegie Mellon University presented a detailed theoretical analysis and simulation of a high order multibit force feedback electro–mechanical ΣΔM for MEMS accelerometers with high Q. However, there was no further update of the related experimental results. Aaltonen et al. [9,10] (2009) at Helsinki University of Technology developed an accelerometer with a high Q (Q > 700) and a 1 kHz fundamental frequency, as shown in Figure 11. The accelerometer was vacuum packaged and operated in a closed-loop control system, which maintained loop stability by damping the Q of the system through electrostatic force feedback. The accelerometer reached a resolution of 300 ng/√Hz (at 30 Hz) with a dynamic range of ±1.5 g and a signal bandwidth of 300 Hz. Xu et al. [11] (2015) at Harbin Institute of Technology developed a closed-loop switched-capacitor ΣΔM CMOS interface circuit for a micromechanical capacitive accelerometer with a noise floor of lower than 200 ng/√Hz (at 100 Hz) and a dynamic range of ±1.2 g. The control loop was implemented in a distributed feedback and feedforward topology, dampening the high Q sensing element by the application of an electrostatic feedback force and phase compensation, which introduced an extra zero to stabilize the loop.

The company Honeywell [54] developed a micromachined accelerometer (QA-3000) using a suspension and proof mass made of quartz, which has a lower material damping compared with silicon and can be used to develop devices with high Q. The small proof mass and the high spring constant of the suspension were compensated significantly by high Q, yielding a noise floor lower than 1 µg/√Hz, a bandwidth larger than 300 Hz as well as a full-scale range of ±60 g.

High Q reduces the *TNEA* of a MEMS accelerometer. However, for a MEMS accelerometer with high Q, the requirements of a closed-loop system control and vacuum packaging increase their complexity and cost, as well as power consumption. Additionally, electronic components from the closed-loop system introduce more electronic noise and thus increase the overall noise floor of the sensor, which may, to some extent, compromise the benefit of low *TNEA* due to high Q. Thus, such a type of accelerometer is only used for specialized applications.

## 5. MEMS Accelerometers with a Low Noise Interface Circuit

Typically, a MEMS accelerometer generates a proof mass displacement in response to an acceleration input, which needs to be transferred into an electronic signal through an interface circuit. However, the noise introduced by those circuits limits the resolution of the MEMS accelerometer. Thus, designers tried to optimize the interface circuit to improve the resolution of MEMS accelerometers.

Si-Ware System (now part of Goodix Technology) [26] developed different ASICs, such as SWS1110, SWS61111 and SWS1120, as well as SWS1130, for capacitive MEMS inertial sensors with an input circuit noise of less than 50 zF/√Hz. The SWS1110 is a configurable ASIC, in which its front-end parameters can be adjusted to work with various inertial sensors in open-loop or closed-loop modes.

**Figure 12:**
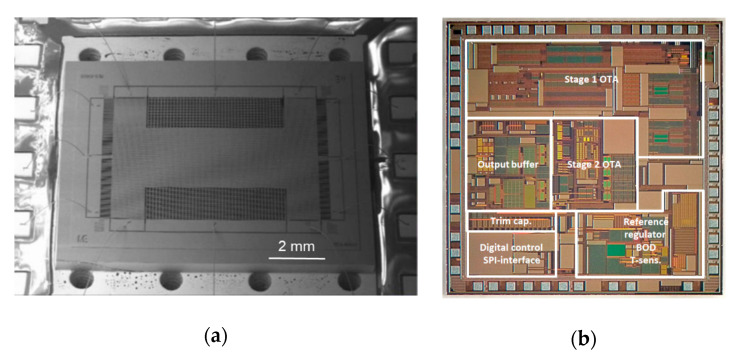
Images of the MEMS accelerometer and the readout ASIC developed by Utz et al. at Fraunhofer Institute for Microelectronic Circuits and Systems [55]: (**a**) SEM image of the MEMS accelerometer; (**b**) photograph of the readout ASIC.

Utz et al. [55] (2018) at the Fraunhofer Institute for Microelectronic Circuits and Systems also developed a capacitance to voltage ASIC with an input noise of 50 zF/√Hz within a bandwidth of 10 Hz to 10 kHz, as shown in Figure 12. The core of the readout circuit was a two-stage, fully differential, chopper-stabilized amplifier in which a chopping frequency of 833 kHz was selected to reduce the low frequency noise, mainly due to flicker noise. With this interface circuit, a conventional MEMS accelerometer with large proof mass achieved a noise floor of 216 ng/√Hz (at 30–40 Hz) and a large bandwidth of 5 kHz. This MEMS accelerometer operated in open-loop mode at atmospheric pressure, hence it did not require a closed-loop control system, reducing the complexity of the system and cost for vacuum packaging.

**Figure 13:**
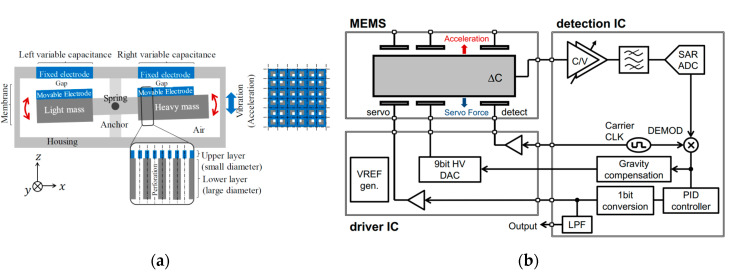
Schematic diagrams of the MEMS accelerometer and the closed-loop system and its closed-loop control system developed by Kamada and Furubayashi et al. at Hitachi [56,57]: (**a**) teeter-totter structure of the MEMS accelerometer with perforations; (**b**) overall block diagram of the closed-loop control system of the MEMS accelerometer.

Kamada and Furubayashi et al. [56,57] (2019) at Hitachi developed a capacitive MEMS accelerometer with an asymmetric so-called “teeter-totter structure” in a getter-free vacuum ceramic package, which reached a noise floor lower than 30 ng/√Hz (at 10–300 Hz) and a dynamic range of 116 dB with a power consumption of 20 mW. It was intended for large sensor network applications, as shown in Figure 13. Figure 13a shows the teeter-totter structure, consisting of a proof mass comprising a heavy mass on one side and a light mass on the other side. The proof mass was suspended with four torsional springs. Pick-off capacitors were formed between the fixed and the moveable electrodes on the proof mass surface. In this configuration, the weight imbalance between the heavy mass and the light mass made the two masses vibrate in opposite directions, resulting in an output capacitance change under a *z*-axis acceleration. To achieve high resolution, different noise sources were addressed by different means. To generate high Q and reduce the Brownian noise, specially designed two-sized perforations were fabricated in the device and substrate layers of the proof mass, in which the upper plate had smaller diameter holes and the lower plate had large ones, as shown in Figure 13a [58]. The Q of the device was 3400 in a getter-less vacuum ceramic package with a pressure of about 6–10 Pa. The 1/f noise of the analog chain was shifted out of the band by modulating ΔC with a 500 kHz pulse applied to the detection capacitors through the driver capacitors. Then, the modulated ΔC signal was demodulated before the proportional–integral–derivative (PID) controller. High-voltage (HV) MOS transistors with channel lengths of 8 μm and widths of several tens of millimeters were adopted for the input differential pair of the voltage-follower to reduce 1/f noise. A PID controller suppressed the in-band 1-bit quantization noise. A digital bandpass ΣΔM modulator created a notch in the quantization noise between 20 and 30 kHz. By isolating the servo- and gravity-compensation capacitors from the op-amp input with the insulation layers, the thermal noise of the front C/V amplifier was suppressed. The servo-signal leakage was still present due to the residual mismatch of the two parasitic capacitances, although it was inherently blocked by an isolation structure. The leakage signal was subtracted from the ADC output by the 1-bit servo-signal after being shaped by a finite impulse response (FIR) filter, whose magnitude of leakage and tap coefficients were chosen by a least mean square (LMS) algorithm. Besides, noise was generated by the coupling between the MEMS higher-order resonant modes and the high-frequency components of the 1-bit quantization noise. To reduce this noise, a digital bandpass delta–sigma modulator (DBPDSM) was used to generate the 1-bit servo-signal instead of the 1-bit quantizer alone, since the former could create a high-frequency notch while preserving the low-frequency signal components. By tuning a parameter of the DBPDSMT, the position of the notch was placed in the region where the higher-order resonances are densely located.

In general, an ASIC interface circuit has lower noise and lower power consumption, as well as smaller size and lower cost for high volume markets compared to a printed circuit board (PCB) interface circuit. Thus, the integration of an ASIC interface circuit with a MEMS accelerometer to achieve a low noise floor has become standard nowadays. However, it requires specialized expertise and often requires several years of development time. Therefore, a PCB interface circuit is preferable for prototype testing.

## 6. MEMS Accelerometers with Signal Readout Methods of Higher Scale Factor

### 6.1. MEMS Accelerometers with High Aspect Ratio Capacitive Gaps

The fabrication of most high precision capacitive MEMS accelerometers relies on DRIE [59]. For the fabrication, the aspect-ratio of the etched trenches and the profile angle are the most critical parameters, since they are directly related to the performance of the fabricated devices. However, footing and lag effects are inherent to the DRIE process due to the decreased active etchant species at the bottom of the trench [60]. These phenomena are problematic for high aspect ratio structures, and hence, for the realization of high-resolution accelerometers.

**Figure 14:**
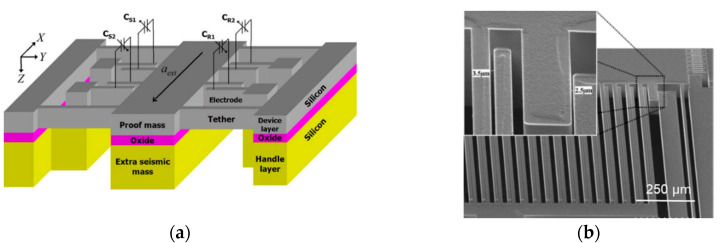
Schematic diagrams of the capacitive MEMS accelerometer with high AR capacitive gap values developed by Abdolvand et al. at Georgia Institute of Technology [62]: (**a**) the MEMS capacitive accelerometer; (**b**) highest gap aspect ratio of 40:1 at the shock-stop gaps.

Ayazi and Najafi [61] (2000) developed a single wafer, all-silicon, high aspect-ratio multi-layer polysilicon micromachining technology that combines the deep dry etching of silicon with conventional surface micromachining. It realized an aspect ratio (AR) of 100:1 and sub-micron gaps with tens to hundreds of microns of thickness. Abdolvand and Ayazi et al. [62] (2007) at the Georgia Institute of Technology developed a capacitive MEMS accelerometer with capacitive gaps with high AR values as shown in Figure 14. The AR was greater than 40:1 and was achieved by depositing a layer of polysilicon on the sidewall of the dry etched gaps. Using this method, on the 100 μm device layer of a silicon on insulator (SOI) wafer, the fabricated gaps of the stoppers, sensing and feedback combs were 2.5 μm (AR 40:1), 4–5 μm (AR 20–25:1) and 3.5 μm (AR 29:1), respectively. The proof mass was significantly increased by leaving the device layer attached to the handle layer, thus making use of the full wafer thickness. Additionally, without requiring a wet-etching step, a compliant suspension with a stiffness of 60 N/m could be fabricated to further improve the sensitivity of the accelerometers. This capacitive MEMS accelerometer resulted in a noise floor of 213 ng/√Hz (at 2 Hz), a bandwidth of 200 Hz, a sensitivity of 35 pF/g and a bias instability of 8 µg (for 3 h).

High AR allows for the realization of MEMS accelerometers with both large proof masses and small capacitive gaps, increasing their sensitivity and suppressing mechanical noise. The related fabrication process is more straightforward compared with other MEMS fabrication processes that realize a large proof mass. However, this requires considerable efforts in the fabrication process development to realize uniform trenches with smooth sidewalls.

### 6.2. Optical MEMS Accelerometers

Capacitive MEMS accelerometers have some inevitable limits due to various noise sources [7], preventing them from achieving even higher sensitivities and resolution. The combination between an optical sensing scheme and MEMS accelerometers may offer ways to overcome some of these limitations.

**Figure 15:**
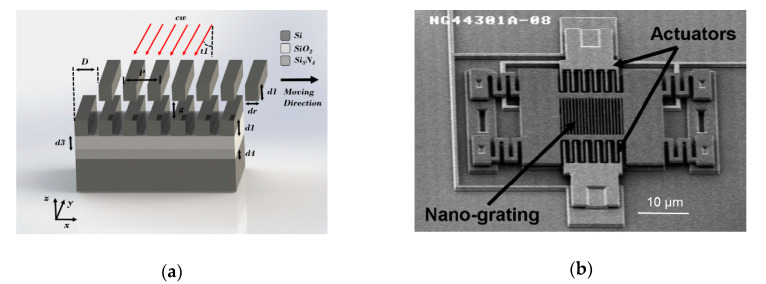
Schematic diagrams of the sub-wavelength gratings and the optical MEMS accelerometer developed by Carr and Keeler et al. at Sandia National Laboratory and Symphony Acoustics [69,70,71]: (**a**) cross section of laterally deformable sub-wavelength grating ([Reprinted] with permission from ref [65] © The Optical Society); (**b**) SEM of fabricated optical MEMS accelerometer.

Wood’s anomaly was first discovered in 1902, which manifests itself as a considerable increase or decrease in the intensity of reflective waves from a grating due to the small variation of the grating structure [63,64,65,66]. Since 2003, Krishnamoorthy and Carr at the Sandia National Laboratory and Symphony Acoustics used this effect to measure small motion [67,68,69,70,71]. Their work was underpinned with rigorous analysis [67], experimental verification [68] as well as an application to an optical accelerometer [69,70,71]. Their integrated sub-wavelength optical nano-grating MEMS accelerometers demonstrated a sensitivity of 590 V/g and a noise floor of 17 ng/√Hz (at 1 Hz). The principle of the device is shown in Figure 15. The two-layer optical gratings were defined in two vertically offset silicon layers suspended in air and separated by an air gap. The upper grating was designed to be moved laterally, while the bottom grating was attached to the silicon substrate. It was first oxidized with a 0.6 μm layer of silicon dioxide and then coated with 0.8 μm low stress silicon nitride to form an antireflective layer.

Hall et al. [72,73] (2008) at the Sandia National Laboratory collaborated with Bicen et al. at the Georgia Institute of Technology to study displacement detectors based on grating interference diffraction and optical MEMS accelerometers. They integrated an electrostatic force excitation mechanism in the accelerometer. A surface-emitting laser (VCSEL) was used as a light source and integrated an ASIC circuit with the accelerometer. This not only allowed the optical MEMS accelerometer to operate at the most sensitive position, but also adjusted the mechanical properties of the micromechanical sensitive structure to regulate the performance of the optical MEMS accelerometer. Its mechanical thermal noise was 43.7 ng/√Hz, but was limited by the test environment (the total inherent noise floor was 24.4 µg/√Hz (at 1 kHz)). Williams et al. [74,75,76] (2014) at the University of Texas at Austin and Silicon Audio developed a MEMS accelerometer based on the micro-grating sensor reported in [73]. It had a closed-loop feedback control and a size of ten cubic centimeters. The bandwidth was from 0.005–1.5 kHz, the dynamic range was 172 dB, the sensitivity was up to 60 V/g and the noise floor was lower than 3 ng/√Hz between 0.1 Hz and 100 Hz (0.5 ng/√Hz at 10 Hz). It could operate in a temperature range between −35 °C and 75 °C.

**Figure 16:**
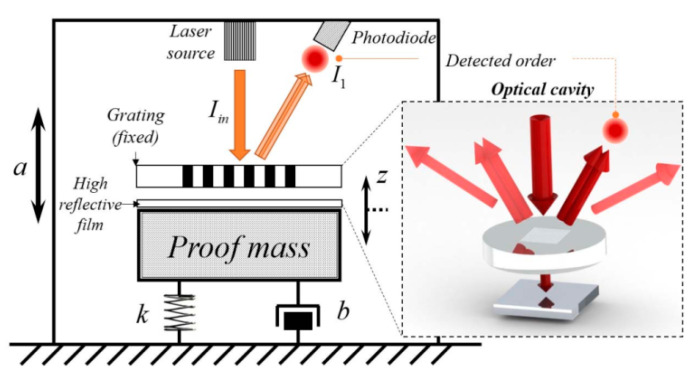
Schematic diagram of the single axis out-of-plane optical MEMS accelerometer developed by Lu et al. at Zhejiang University [77].

Lu et al. [77] (2017) at Zhejiang University developed an optical MEMS accelerometer, similar to the design of the Sandia National Laboratory [73], by measuring the acceleration through the change of the interference pattern produced by two gratings, as shown in Figure 16. One grating was suspended above the proof mass and the other was on the surface of the proof mass to form a cavity between the two gratings [78,79,80]. With light illuminated on the top grating by a laser source, part of the light was transmitted to the bottom grating and reflected to produce an interference pattern, which was detected by a photodiode. The proof mass moved up or down with acceleration, which led to a change in the cavity and relative change in the interference pattern. Thus, the input acceleration could be derived from the output signal of the photodiode. Experimental results showed that the optical MEMS accelerometer achieved an acceleration sensitivity of 2485 V/g with a resonant frequency of 34.5 Hz and a noise floor of 186 ng/√Hz.

Guzman Cervantes et al. [81] (2013) at the National Institute of Standards and Technology designed an optical MEMS accelerometer based on Fabry–Pérot (FP) sensing [82,83,84], which combined a monolithic fused-silica oscillator and a fiber-optic micro-cavity into an absolute reference accelerometer. A noise floor of 100 ng/√Hz over a bandwidth of 10 kHz was achieved above 1.5 kHz. A noise floor of better than 10 ng/√Hz over a bandwidth of 2 kHz was achieved above 9 kHz. Bao et al. [85] (2016) at the National Institute of Standards and Technology developed a FP interferometer-based optical MEMS accelerometer with a sub-µg/√Hz noise floor. The optical MEMS accelerometer had a hemispherical optical cavity of high finesse, which was locked by a tunable laser and thereby demonstrated the possibility for the closed-loop operation of the accelerometer. Zhao et al. [86] (2020) at Xi’an Jiaotong University created an optical MEMS accelerometer based on FP with a sensitivity of 183.8 V/g, a bandwidth of 29.3 Hz as well as a noise floor of 42.4 ng/√Hz. It consisted of a G-shaped mass-spring structure sensing chip, laser diode, cube beam splitter and photo translating system, which were integrated by a 3D printed sensor package.

Fourguette et al. [87,88] (2011) at the Michigan Aerospace Corporation created an optical MEMS-based accelerometer with a noise floor of 10 ng/√Hz based on Whispering Gallery modes, which shifted a morphology-dependent optical resonance in small dielectric spheres (<1 mm in diameter), making the transducer highly sensitive to force (<10^−9^ N). The optical MEMS-based accelerometer comprised a proof mass suspended by a spring assembly and a polymer microsphere positioned between the proof mass and the accelerometer base. Besides, an optical fiber was used for coupling infrared light into the equatorial region of the microsphere. As the proof mass compressed the polymer microsphere under acceleration, the morphological deformation of the microsphere shifted the naturally occurring optical resonances in the sphere. Hence the system was capable of detecting an acceleration as small as 10 ng.

Li et al. [89] (2016) at Wuhan University of Technology developed a Fiber Bragg Gratings (FBGs) fiber-optic interferometric optical micro-vibration sensor, in which, for the first time, the fiber itself was used as a mechanical suspension element and not only as a strain sensor. Duo et al. [90] (2018) at Peking University and Shenzhen University developed a FBGs fiber-optic interferometric optical MEMS accelerometer with a noise floor of about 312 ng/√Hz (at 100 Hz). The self-suppressing common-mode noise scheme proposed effectively reduced the volume and cost of the accelerometer and improved the signal-to-noise ratio of the output signal.

Based on work using grating light valves [91], Loh et al. [92] (2002) at the Massachusetts Institute of Technology used a large bulk-micromachined proof mass and an interferometric sensor to develop an optical MEMS accelerometer with a noise floor of 40 ng/√Hz (at 40 Hz), a bandwidth ranging from 80 Hz to 1 kHz as well as a dynamic range of 85 dB at 40 Hz. The interferometer comprised one group of comb fingers fixed on the outer frame and another group attached to the proof mass. The two comb finger groups formed the grating structure, which was illuminated by a laser and reflected a series of light beams. The intensity of the reflected beams changed with the relative out-of-plane displacement between the proof mass and the outer frame.

Jaksic et al. (2004) [93] at the Institute of Microelectronic Technologies and the company Single Crystals first proposed an optical MEMS accelerometer based on photonic crystal waveguides. One- or two-dimensional photonic crystal waveguides were used to detect stress or strain in mechanically sensitive structures. This type of optical MEMS accelerometer had the advantages of all-optical signal communication, relatively simple fabrication and high integration with the chip, but the first prototype was not very accurate. Krause et al. [94] (2012) at the California Institute of Technology designed an optical MEMS accelerometer based on a photonic crystal nanocavity displacement detection unit [95]. A fixed beam with micropores and the upper end of the proof mass (also with micropores) formed a photonic crystal nanocavity with a zipper shape. The nanocavity acted as an optical displacement sensor when the mass was displaced by acceleration. When the gap of the cavity changed, the transmittance of different wavelengths (mode) changed, and the input acceleration could be calculated by detecting the transmitted light intensity. This optical MEMS accelerometer had a high optical Q of 1.4 × 10^6^ in a vacuum and a bandwidth greater than 20 kHz, as well as a dynamic range greater than 40 dB. The displacement measurement accuracy was better than 4 fm/√Hz. Although the accelerometer only reached a noise floor of 10 µg/√Hz (at 5–25 kHz), with the introduction of a higher resolution interface circuit, the noise floor could be reduced to sub-µg/√Hz.

Flores et al. [96] (2016) at the University of California presented an optical MEMS accelerometer with a DC noise floor of 196 ng/√Hz [88]. This MEMS accelerometer made use of an ultrasensitive displacement readout using a slot-type photonic crystal cavity with an optical gradient force transduction, which formed localized optical resonant modes with shifted and perturbed lattice holes [97,98].

Miao and Aksyuk et al. [99] (2012) at the National Institute of Standards and Technology developed a cavity optomechanical sensing system with a displacement sensitivity of 4.6 fm/√Hz and a force sensitivity of 53 aN/√Hz with only 250 nW optical power. The system had a high-quality-factor interferometric readout, in which an optomechanical coupling could be electrically tuned by two orders of magnitude and the mechanical transfer function was adjustable via a feedback control. Cold damping feedback was used to reduce the thermal mechanical vibration of the sensor by three orders of magnitude and to broaden the sensor bandwidth, by approximately the same factor, to more than twice the fundamental frequency of ≈40 kHz. The readout sensitivity approaching the standard quantum limit was combined with MEMS actuation in a fully integrated, compact, low-power, stable system compatible with Si batch fabrication and electronics integration. Though they have not built an accelerometer, this technology is promising to achieve an optical MEMS accelerometer with a sub-ug/√Hz noise floor.

Optical MEMS accelerometers have recently seen significant progress, as discussed above, thanks to their high sensitivity of optical sensing schemes, but they are still limited in terms of integration potential and compatibility with harsh environmental applications.

#### 6.2.1. Resonant MEMS Accelerometers with Frequency Readout

**Figure 17:**
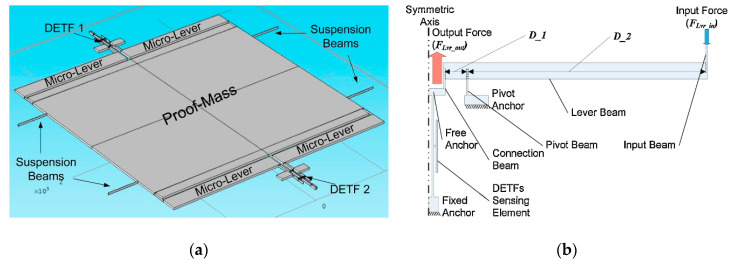
Schematic diagrams of the resonant MEMS accelerometer developed by Zou and Seshia at Cambridge University [101]: (**a**) the resonant MEMS accelerometer comprising four single stage force amplification mechanisms; (**b**) a single-stage force amplification mechanism.

As resonant MEMS accelerometers sense acceleration input through the force-induced frequency shift of a double-ended tuning fork (DETF), typically, they are less susceptible to external environmental effects (e.g., temperature fluctuations), especially at low frequencies. Zou and Seshia [100] (2015) at Cambridge University developed a vacuum packaged resonant MEMS accelerometer with a noise floor of 144 ng/√Hz (at <1–50 Hz), which benefited from a large proof mass and four single-stage force amplification mechanisms [101], as shown in Figure 17. With the force amplification mechanism, the inertial force input on the proof mass due to acceleration input was amplified by a leverage structure without introducing noise to a first-order approximation. Then, the amplified force was transmitted to two DETFs, shifting its resonant frequency. Since the two DETFs were situated on either side of a proof mass, they experienced a differential axial force due to input gravitational acceleration resulting in a differential frequency shift signal. The accelerometer featured a scale factor of 960 Hz/m/s^2^ and a dynamic range of ±0.5 m/s^2^. Pandit and Seshia et al. [102] (2019) at Cambridge University improved the noise floor of Zou et al.’s design to 18 ng/√Hz (at 0.25–4 Hz) with a bias stability of 18 ng and a bandwidth of 5 Hz. The low noise floor was achieved by post-processing the output signal to reduce the influence of temperature on the frequency shift of the DETFs (e.g., due to the difference in the temperature coefficients of the two DETFs and temperature gradients across the chip).

**Figure 18:**
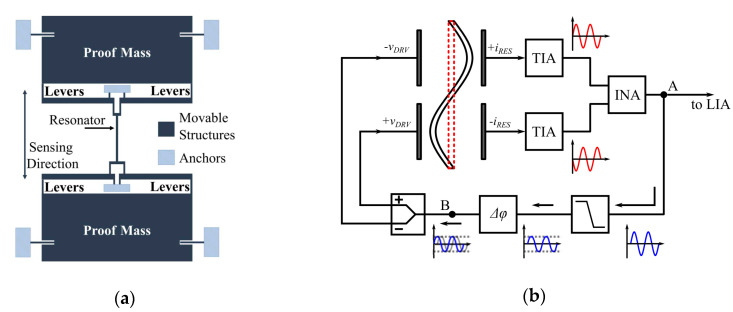
Schematic diagrams of the resonant MEMS accelerometer and the closed-loop circuit developed by Zhao and Seshia et al. at Cambridge University [103]: (**a**) resonant MEMS accelerometer comprising a single DETF; (**b**) closed-loop circuit.

Zhao and Seshia et al. [103] (2019) at Cambridge University developed a resonant MEMS accelerometer with a noise floor of 98 ng/√Hz (at 1 Hz), a bias stability of 56 ng and a bandwidth of 5 Hz, which comprised a single DETF, sandwiched between two proof masses, as shown in Figure 18a. The single DETF operated in the second lateral transverse mode though a differential driving configuration, as shown in Figure 18b, which led to a higher critical linear amplitude and thus a higher sensitivity. Besides, due to the single DETF design with inverting levers, oscillator cross-talk and undesired locking could be avoided, which commonly exist in a conventional two DETF design [100,102] due to the mechanical coupling of the two DETFs.

Another resonant MEMS accelerometer was developed by Yin et al. [104] (2017) at Tinghua University. It comprised a single DETF with a noise floor of 380 ng/√Hz, a bandwidth of 2.7 Hz and a full scale range of ±15 g. They analyzed various frequency noise sources in detail, including nonlinear vibration noise, frequency measurement noise, vibration amplitude-independent noise and subsequently optimized the vibration amplitude of the DETF to minimize the noise floor. Additionally, the device was vacuum packaged to implement the DETF with a high Q up to 350,000, which increased the vibration amplitude and sensitivity. Besides, the micro-lever and the DETF as well as the bearing beam, were optimized to produce a high sensitivity of 244.15 Hz/g, further reducing the noise floor.

Shin and Kenny et al. [105] (2017) at Stanford University developed a resonant MEMS accelerometer across a wide temperature range, as well as with excellent stability. The accelerometer had a bias instability of 160 ng at a 21 s integration time and a scale factor of 427 Hz/g. Experimental results showed a scale factor stability of 0.38% over the temperature range from −20 °C to 80 °C, which was in part attributed to the close proximity of the resonators, minimizing any potential temperature gradient between the beams. The one anchor design improved the insensitivity to package stress.

The resonant MEMS accelerometers mentioned above are all made of silicon, and the other resonant MEMS accelerometers are fabricated from quartz. ONERA [106,107,108] has been a pioneer in developing quartz resonant MEMS accelerometers. ONERA developed a single vibrating beam instead of a DETF for quartz resonant MEMS accelerometers, designated as VIA and DVIA [107]. The main novelty of these designs was the insulating system (the frame and the two links), connecting the active part to the mounting area, which insulated the vibrating beam from thermal stresses, improving the frequency stability and the temperature behavior. The final noise floor of the VIA and DVIA type quartz resonant MEMS accelerometers was 5 µg/√Hz (at 1 Hz) with a bandwidth larger than 1000 Hz and a full scale range of ±100 g. A more compact design allowing a higher natural frequency and a larger proof mass can further improve the noise floor of such quartz resonant MEMS accelerometers to a potentially sub-µg/√Hz level.

Resonant MEMS accelerometers transduce the inertial force on a proof mass as a shift in the natural frequency of a resonator, offering high sensitivity without compromising the bandwidth or size, as in conventional MEMS accelerometers [46]. Besides, the resolution of frequency readout techniques is higher than that of conventional amplitude readout methods, which further reduces the noise floor. Furthermore, the quasi-digital signal of a frequency readout signal is convenient for integration with other devices. However, resonant MEMS accelerometers suffer from effects such as temperature sensitivity (requiring temperature compensation techniques), the requirement for vacuum packaging, as well as the complexity of closed-loop readout interfaces.

#### 6.2.2. Resonant MEMS Accelerometers with Amplitude Ratio Readout (Based on Mode Localization Effect)

**Figure 19:**
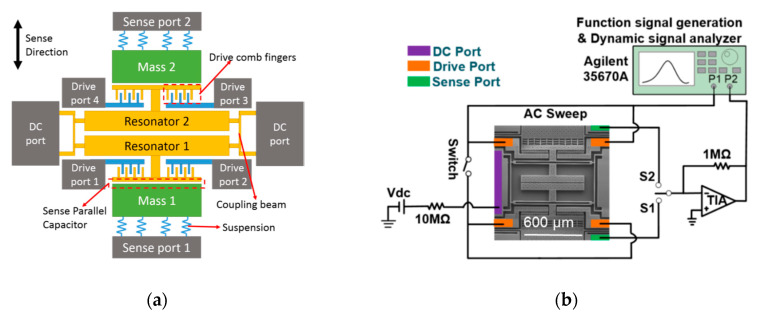
Schematic diagrams of 2DoF WCR accelerometer and its open-loop measurement setup developed by Zhang et al. at Northwestern Polytechnical University [110]: (**a**) a 2DoF WCR accelerometer; (**b**) open-loop frequency response measurement setup.

Recently, an alternative sensing approach called the mode localization effect has been proposed [109]. Mode localization can be described as a small symmetry-breaking perturbation to a periodical oscillating system with two (or more) symmetric resonant units. This effect leads to energy confinement on one resonator, so-called mode localization and curve veering. It has been shown that the sensitivity is improved by orders of magnitude compared to conventional frequency modulation in devices of similar sizes and fundamental resonant frequencies. The premise of sensitivity enhancement is to choose the eigenstates or amplitude ratio as the output metrics for the mode localization-based resonant sensors, but not the change in resonance frequency. A few MEMS resonant accelerometers have already been developed based on this effect. When acceleration acts on a proof mass, differential electrostatic stiffness perturbations will be applied to weakly coupling resonators (WCR). The WCR is used to realize a two (or higher) degree-of-freedom (DoF) resonator system. The stiffness perturbation causes mode localization, and thus mode shape changes. Acceleration can be sensed by measuring the amplitude shift of the resonant masses. Zhang et al. [110] (2016) at Northwestern Polytechnical University (NPU) developed a 2DoF WCR MEMS accelerometer with an amplitude ratio sensitivity of about 312,162 ppm/g, as shown in Figure 19. Pandit and Seshia et al. [111] (2018) at Cambridge University developed a closed-loop 2DoF WCR accelerometer with a bias stability of 7 µg, by combining the mode localization effect with a force amplification mechanism. Pandit and Seshia et al. [111,112] (2019) further implemented a two 2DoF WCR into the accelerometer design and realized a sensing system based on differential mode localization. This accelerometer reached a bias stability of 2.96 µg, a noise floor of 3 µg/√Hz and a bandwidth of 350 Hz. Since a 3DoF WCR had an even higher amplitude ratio sensitivity [113], Kang et al. [114] (2017) at NPU developed a 3DoF WCR accelerometer, whose absolute sensitivity was improved by 349% compared to the 2DoF WCR accelerometer, described in [110,111]. Kang et al. [115] (2018) also implemented a closed-loop system for the 3DoF WCR accelerometer, reaching a noise floor of 1.1 µg/√Hz and a bias stability of 157 µg.

Zhao and Seshia et al. [116] (2019) at Cambridge University developed a closed-loop 2DoF WCR accelerometer with a bias stability of 680 ng/√Hz (in a frequency band of 0.5–3 Hz), by introducing parametric modulation. Utilizing this technique, they showed that the scale factor of the accelerometer could be enhanced by a factor of 188, and a 25-fold improvement in sensor resolution was demonstrated compared to their earlier work.

Existing WCR accelerometers have not yet demonstrated a noise floor better than the conventional resonant MEMS accelerometers, since amplitude readout methods tend to have a lower resolution compared to frequency readout methods. However, WCR accelerometers exhibit better bias stability due to their common mode rejection properties (e.g., for temperature fluctuations) [117]. Moreover, the aforementioned recent work considerably improved the understanding of various fundamental drift and noise mechanisms affecting the resolution. This underpinned the potential of future WCR accelerometers with a resolution superior to conventional resonant MEMS accelerometers. However, the requirement for vacuum packaging and the complexity of closed-loop readout interfaces are again shortcomings of WCR accelerometers.

#### 6.2.3. MEMS Tunneling Accelerometers

**Figure 20:**
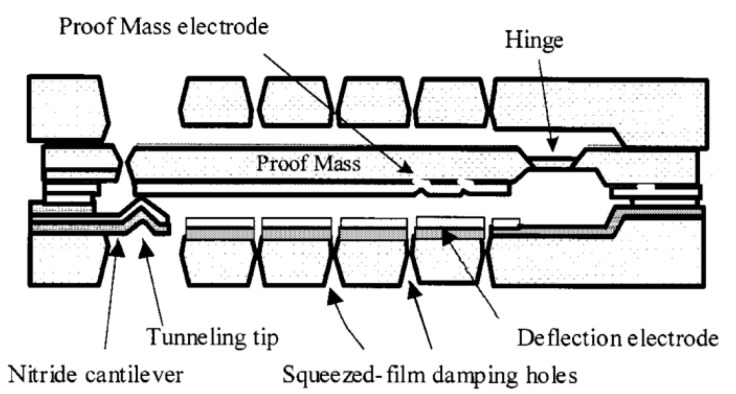
Schematic diagram of a MEMS tunneling accelerometer developed by Liu and Kenny at Stanford University [118].

Liu and Kenny [118] (2001) at Stanford University developed an accelerometer with high sensitivity and wide bandwidth based on the electron tunneling effect. As shown in Figure 20, it consisted of a proof mass, a tunneling tip and a proof mass electrode, as well as a deflection electrode. The gap between the proof mass and the tunneling tip was kept at the tunneling position (<1 nm distance from the proof mass) by an electrostatic force generated by a servo mechanism, even when the proof mass was deflected under acceleration input. Thus, the output control signal changed with the input acceleration. Experimental results revealed that the MEMS tunneling accelerometer achieved a noise floor of 20 ng/√Hz (at 10–1000 Hz) over a bandwidth between 5 Hz to 1.5 kHz with a closed-loop dynamic range of over 90 dB.

The high sensitivity of the tunneling sensing scheme leads to sensitive MEMS accelerometers; this reduces the requirement for a large proof mass or a small spring constant to achieve a low noise floor. Therefore, a relatively high bandwidth of the accelerometer can be achieved. However, a MEMS tunneling accelerometer with a noise floor of sub-µg/√Hz is required to reliably maintain a small gap (1 nm) between the tunneling tip and the proof mass. Such a small gap is difficult to be fabricated. Due to fabrication tolerances, the repeatability and uniformity of different devices cannot easily be ensured. Furthermore, it requires precise closed-loop control to prevent the contact of the tip and the proof mass under a large acceleration input. This limits the applications in harsh environments. Additionally, the noise floor at low frequencies (<10 Hz) of tunneling accelerometers is determined by 1/f noise.

#### 6.2.4. MEMS-Based Electrochemical Accelerometers

**Figure 21:**
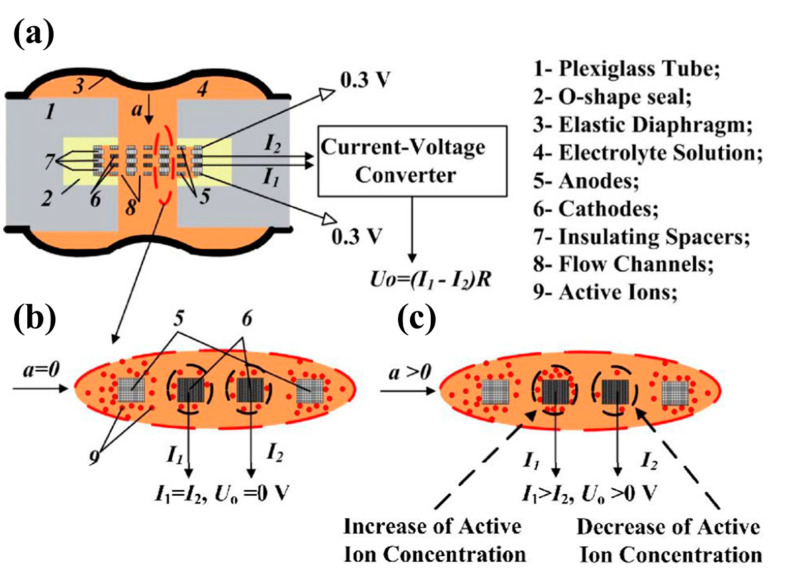
Schematic diagrams of an electrochemical accelerometer developed by Deng et al. at the Chinese Academy of Sciences [122]: (**a**) device; (**b**) with no acceleration input, no output voltage signal is generated as active ion concentrations surrounding both cathodes are equal; (**c**) under acceleration, a voltage output is generated as ion concentration around one cathode increases while it decreases at the other electrode.

As opposed to other accelerometers with a solid inertial mass, electrochemical accelerometers use a liquid solution as an inertial mass [119], the movement of which is converted to a variation of output electric currents by a four-electrode sensing unit, which is illustrated in Figure 21. Hua [120] (2014) at Arizona State University developed an electrochemical accelerometer with an inherent noise floor of 44 ng//√H (at 1 Hz). By using microfabrication techniques, the inter-electrode spacing of the electrochemical accelerometer was reduced to 1 μm, which improved the sensitivity of the fabricated device to above 3000 V/(m/s^2^) for an operating bias of 600 mV and an input acceleration of 400 μg at 0.32 Hz. The hydrodynamic resistance was lowered by increasing the number of channels and improving the noise floor. Liang and Hua et al. [121] (2016) at Arizona State University and the Moscow Institute of Physics and Technology developed an electrochemical accelerometer with a noise floor of 17.8 ng/√Hz (at 1.2 Hz) and a sensitivity of 250 V/g. A SOI-based micro-fabrication process was employed to reduce the hydraulic impedance of microfluidic channels, which increased the sensitivity and lowered the noise floor.

Since thinner insulating spacers result in higher device sensitivity, Deng et al. [122] (2014) at the Chinese Academy of Sciences further reduced the thickness of the insulating spacer and developed a MEMS-based electrochemical accelerometer with a noise floor of 10 ng/√Hz (at 1 Hz). The electrochemical accelerometer had a maximum acceleration input of 10 mg, as well as a bandwidth of 0.2–5 Hz, as illustrated in Figure 21 [122]. Based on Huang et al.’s work [123], the insulating spacer and electrodes were fabricated by Deng et al. [124] (2016) on the same silicon wafer, reducing fabrication complexity. As a result, a MEMS-based electrochemical accelerometer with a noise floor of 3.2 ng/√Hz (at 0.02 Hz) was demonstrated.

Different from other MEMS accelerometers, electrochemical accelerometers use a liquid solution as their proof mass, which facilitates easy deployment and transportation—especially in harsh environments—low noise floor at low frequencies and independence on the installation angle. However, the size of a MEMS-based electrochemical accelerometer is larger than a conventional MEMS accelerometer and its bandwidth is small (a bandwidth of 0.2–5 Hz in Deng et al.’s work [122]).

#### 6.2.5. Electrostatically Levitated MEMS Accelerometers

Electrostatically levitating a proof mass eliminates the need to overcome the elastic restraint of mechanical supports, which theoretically will result in considerably higher sensitivities and less dependence on fabrication tolerances, as well as more flexibility in adjusting the device characteristics of bandwidth and sensitivity without the need to redesign mechanical flexures. A further advantage is the potential for multi-axis sensing with one device. The major obstacle to develop such accelerometers is the complexity of the control loop. The most sensitive electrostatically levitated MEMS accelerometers only reached a noise floor of 3 µg/√Hz (at 0.2–10 Hz), described by Han et al. at Tsinghua University [125] (2015), but it is a promising method to reduce the spring constant of MEMS accelerometers to achieve sub-µg/√Hz noise floor in the future.

**Figure 22:**
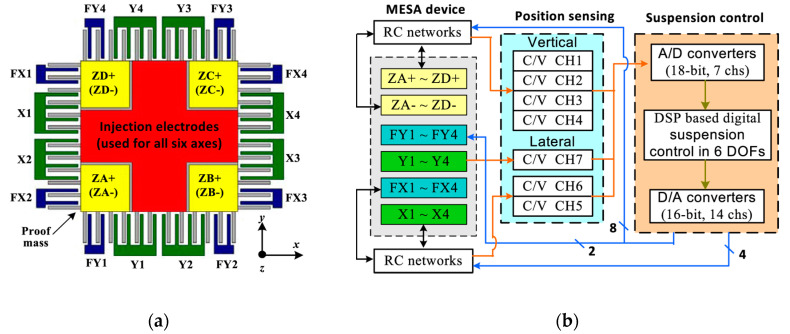
Schematic diagrams of MEMS electrostatically levitated accelerometer electrodes and suspension control loop developed by Han et al. at Tsinghua University [125]: (**a**) top view of device electrode; (**b**) digital suspension control loop.

Houlihan and Kraft [126,127,128] (2002) at Southampton University designed and simulated an electrostatically levitated MEMS accelerometer and developed a related interface circuit. Cui et al. [129] (2011) at Shanghai Jiaotong University designed and fabricated a micromachined electrostatically levitated six-axis accelerometer, experimentally validating the proof of concept. However, none of these publications describe a comprehensive experimental evaluation proving the performance of an electrostatically levitated MEMS accelerometer. Toda et al. [130] (2002) at Ball Semiconductor developed an electrostatically levitated ball used as the proof mass of an accelerometer, achieving a noise floor better than 40 µg/√Hz. It was fabricated by a novel sacrificial etching process utilizing Xenon difluoride gas etching through a gas permeable layer. The ball had a diameter of 1 mm and was suspended by closed-loop controlled electrostatic forces. Of note, 3-axis acceleration sensing could be derived from the magnitude of the voltages for the servo feedback system. Han et al. [125] (2015) at Tsinghua University developed an electrostatically levitated three-axis MEMS accelerometer, which was realized as a glass/silicon/glass wafer-bonded structure, fabricated by a bulk micromachining process. It was reported that this accelerometer had a noise floor of 3 µg/√Hz (at 0.2–10 Hz), a sensitivity of 689 V/g, a full-scale range of ±2.9 mg and a bandwidth from 0.2 to 10 Hz. Its rather complex control system and electrode design are illustrated in Figure 22.

Electrostatically levitated MEMS accelerometers have a potential to achieve a low noise floor, since their electrostatic spring constant theoretically can be made much lower than any mechanical counterparts. However, this type of MEMS accelerometer requires complex levitation and control techniques.

#### 6.2.6. MEMS Accelerometer Using Time Transduction

**Figure 23:**
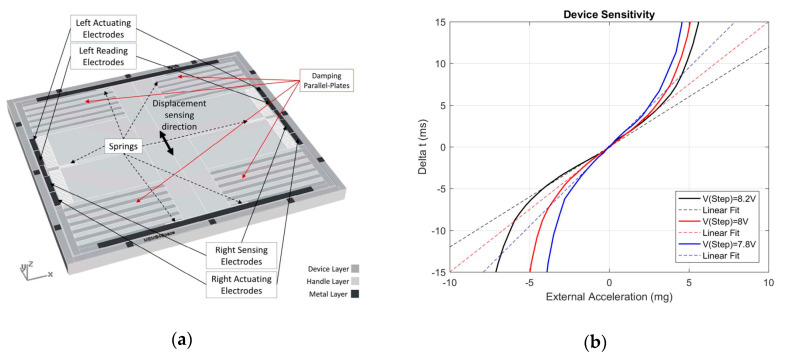
Schematic diagrams of the MEMS accelerometer by Garcia et al. at University of Minho and its sensitivity [132]: (**a**) MEMS accelerometer, (**b**) measured sensitivity.

Pakula and French [131] (2007) at Delft University of Technology developed a MEMS accelerometer based on time transduction. As shown in Figure 23, the MEMS accelerometer was continuously actuated by a differential set of driving electrodes to the electrostatic pull-in state. A differential set of sensing electrodes were used to measure the displacement of the proof mass and detect the time between the rise of the actuation step and the pull-in state. When the MEMS accelerometer was used at specific damping conditions, the pull-in effect created a meta-stable phase for the proof mass displacement. For an acceleration input, the time that the proof mass remains in the metastable phase changes. Such a working principle allows a very high precision since a time measurement is performed rather than direct capacitive transduction. Garcia et al. [132] (2019) at the University of Minho developed a MEMS accelerometer using the pull-in time as a sensing scheme with a sensitivity of 1.9 ns/ng. A large proof mass (126 mg) was fabricated and the squeeze-film damping was increased (Q = 0.53) by using the handle layer of a SOI wafer.

Garcia et al. did not report the noise floor of the MEMS accelerometer, but with its sensitivity of 1.9 ns/ng, such MEMS accelerometers can reach a resolution better than 1 µg if the resolution of the timer is better than 1.9 µs.

## 7. Discussion

**Figure 24:**
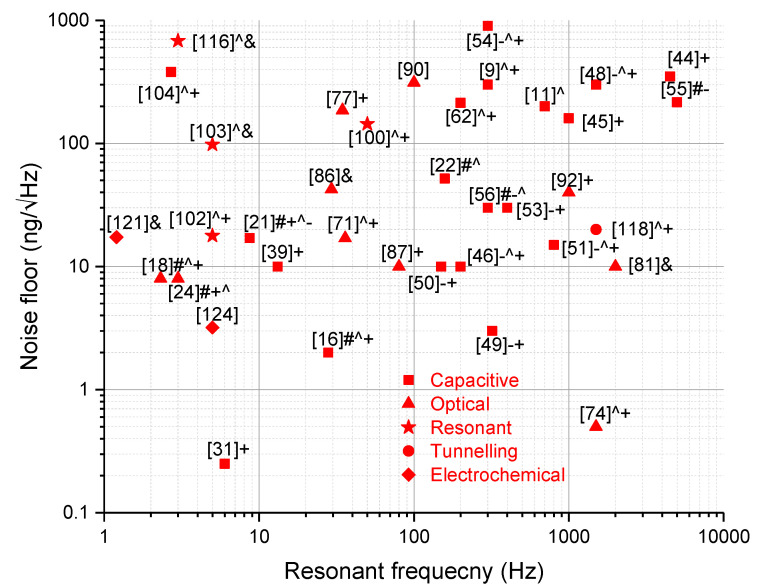
Comparison of micromachined accelerometers with a sub-µg/√Hz noise floor. “+” refers to large proof mass, “#” refers to low spring constant, “–“ refers to low noise interface circuit and “^” refers to high Q.

As can be seen from this review, there are a considerable number of MEMS accelerometers with sub-µg/√Hz noise floors, and these are summarized in Figure 24. Various accelerometers with different sensing schemes and structural designs have been discussed in detail; however, their main characteristics are listed in Table 3 and Table 4. Most of the sensors achieved sub-µg/√Hz noise floor by using different concepts simultaneously (i.e., large proof mass, low spring constant, low-noise electronic interface circuit, etc.).

Since *ENEA* is typically the dominant noise source for MEMS accelerometers, there is currently a focus on improving interface circuits to reduce it. Compared to PCB read-out circuits, ASIC implementations have the advantages of lower power, smaller volume and lower noise, as well as suitability for mass production. Therefore, ASICs have become the standard for micromachined accelerometers. Low frequency noise and bias instability are challenging for seismic measurements. The noise source mainly originates from flicker noise. Some researchers used modulation techniques to suppress flicker noise with a careful choice of the modulation frequency. As for the closed-loop mode of operation, some researchers reduced cross coupling between the sense and feedback electrodes through an electrical isolation layer in the structure, which could be further reduced by an electronic filter algorithm. Other groups used a digital band-pass ΣΔM modulator to create a notch in the profile of the quantization noise to minimize the noise generated by the 1-bit quantization in a digital feedback closed-loop system.

Most MEMS accelerometers achieved low *TNEA* and large scale factor by realizing a large proof mass fabricated by bulk micromachining or by using materials of a higher density. Additionally, some researchers used vacuum encapsulation for a high Q to obtain lower *TNEA* of MEMS accelerometers. Other designs were based on special structures to increase the Q (i.e., perforation structures, replacing gap changing capacitors with area changing capacitors, more channels allowing air to flow, the material of lower energy loss, improving material impurities and defects, reducing anchor loss, suppressing or removing interfering modes). However, high Q can lead to the instability of MEMS accelerometers. To compensate the stability issue, different closed-loop systems were developed to maintain a stable operation. Electro–mechanical ΣΔM modulators were used by many groups to achieve a closed-loop control due to their noise shaping ability and direct digital output. Thus, improving the Q can lead to a lower noise floor, but requires more complex closed-loop circuits, which may introduce additional noise and higher power consumption and may require vacuum packaging process, increasing cost and fabrication complexity. Another approach is to reduce the spring constant of the suspension system to achieve a higher scale factor and a lower resonant frequency, which reduces the *TNEA*. Recently, GAS was used to realize a low overall spring constant; other methods included the electrostatic levitation of the proof mass, as well as using materials of lower Young’s modulus, such as Parylene. GAS is a new type of suspension system which can reduce spring constant to a small value without pushing the fabrication to its limitation. MEMS accelerometers with GAS resulted in higher signal stability compared to other MEMS accelerometers with a sub-µg/√Hz noise floor. As for electrostatic levitation, described prototypes have, to date, not achieved a noise floor below 1 µg/√Hz. However, theoretically, levitated MEMS accelerometers can have a spring constant lower than any mechanical suspension system, making it a very promising suspension scheme to improve the resolution of such sensors below a 1 µg/√Hz noise floor.

Most MEMS accelerometers with a noise floor lower than or equal to 10 ng/√Hz mainly achieved this by improving the mechanical design, most notably using a geometric antispring suspension (GAS) [16,18,24] or by simply increasing the proof mass weight [31,39,46,49,50]. Five MEMS accelerometers reached such a low noise floor used optical sensing schemes, including optical micro-grating sensing [87], morphology-dependent optical resonance sensing [74], optical FP sensing [81] or optical shadow sensor sensing [18,24]. Only one MEMS accelerometer reached such a low noise floor using an electrochemical sensing scheme [122].

As shown in Table 3, commercial MEMS accelerometers [46,48,49,50,51,53,54] achieved a sub-µg/√Hz noise floor mainly through the usage of a large proof mass and a low noise interface circuit and vacuum packaging. Since the performance stability is a critical character of a commercial product, companies prefer to take those mature technologies mentioned above to improve the noise floor of MEMS accelerometers.

## 8. Conclusions

In summary, this review attempts to describe current innovations and issues predominating the field of micromachined accelerometers with a sub-µg/√Hz noise floor by discussing design and sensing schemes, as well as other approaches. As illustrated above, structures, materials, fabrication processes and circuit technologies are being constantly updated to improve resolution and other performance metrics. There are two trends for further developments from our point of view: (i) Further optimizing the structural design of classic spring–mass–damper systems, potentially in combination with new structures made of non-silicon materials requiring novel fabrication techniques. Furthermore, corresponding circuit technologies should be concurrently developed. (ii) Utilizing novel sensing schemes to increase the signal scale factor, improving the SNR and lowering the noise floor. Moreover, as can be seen from this review, many MEMS accelerometers achieved a sub-µg/√Hz noise floor. However, signals at frequencies below 10^−2^ Hz are still challenging and signals down to 10^−4^ Hz in the regime of spatial or temporal gravity surveys require accelerometers with excellent stability for days, weeks or even months. Although MEMS accelerometers are now ubiquitous, few achieve sensitivities that could be considered useful for gravity surveys or future gravitational wave detectors, and none have achieved a sufficient stability to compete with commercial macroscopic accelerometers (with one exception, reported in [17]).

Being a niche and lucrative sector of micromachined sensor development and design, the fabrication of micromachined accelerometers with a sub-µg/√Hz noise floor will result in greater accuracy in the measurement of gravity measurement, resource exploration, seismic monitoring and other applications in the future.

## Figures and Tables

**Table 1:** Typical specifications of MEMS accelerometers on three performance levels, including tactical grade, navigation grade as well as auto control [5].

Parameters	Navigation Grade	Tactical Grade	Consumer Grade
Input range	±1 g	±5 g	±50 g
Noise	<50 μg	<100 μg	<50 mg
Working Frequency	100 Hz	100 Hz	400 Hz
Sensitivity	<100 μg	<200 μg	<50 mg
Nonlinearity	<0.05%	<0.1%	<2%
Max shock input	>10 g	>20 g	>2000 g
Partial axis sensitivity	<0.1%	<0.3%	<5%

**Table 2:** Summary of commercial capacitive MEMS accelerometers with a sub-µg/√Hz noise floor.

Company	Product	Noise Floor	Dynamic Range/Full Scale Range	Bandwidth
HP	---	10 ng/√Hz (at 1–200 Hz)	120 dB	1–200 Hz
Colibrys	SF1500	300 ng/√Hz (at 10–1000 Hz)	117 dB	0–1500 Hz
	SF2005S.A	800 ng/√Hz (at 10–1000 Hz)	111 dB	0–1000 Hz
	SF3000	300 ng/√Hz (at 10–1000 Hz)	120 dB	0–1000 Hz
	SI1000	700 ng/√Hz (at 0.1–100 Hz)	108.5 dB	0–550 Hz
Kinemetrics	EpiSensor ES-T	60 ng/√Hz	±0.25 g	0–200 Hz
	EpiSensor ES-U2	60 ng/√Hz	±0.25 g	0–200 Hz
	EpiSensor 2	3 ng/√Hz (at 1 Hz)	±0.25 g	0–>320 Hz
Reftek	131A	14 ng/√Hz	±3 g	0–500 Hz
	Trimble 147A	10 ng/√Hz	±4 g	0–150 Hz
Sercel	DSU1-508	15 ng/√Hz (at 10–200 Hz)	128 dB	0–800 Hz
	DSU-3	41 ng/√Hz (at 10–200 Hz)	120 dB	0–800 Hz
INOVA	ACCUSEIS SL11	30 ng/√Hz (at 3–400 Hz)	118 dB	3–400 Hz
	VECTORSEIS ML21	40 ng/√Hz (at 3–375 Hz)	118 dB	3–375 Hz

**Table 3:** Summary of micromachined accelerometers with a sub-µg/√Hz noise floor.

Research Group	Noise Floor	Dynamic Range /Full Scale Range	Resonant Frequency	Type
Boom and Kamp et al. [16]	2 ng/√Hz (at 28.1 Hz)	--	3–28.1 Hz	#+^
Middlemiss et al. [18]	8 ng/√Hz (at 1 Hz)	--	2.31 Hz	#+^
Zhang et al. [22]	51.8 ng/√Hz (at 1 Hz)	--	158 Hz	#^
Tang et al. [24]	8 ng/√Hz (at 1 Hz)	+8 mg	0.5–3 Hz	#+^
Pike et al. [31]	0.25 ng/√Hz (at 0.1–10 Hz)	--	6 Hz	+
Wu et al. [39]	10–50 ng/√Hz (at 1 Hz)	±1.4 g	13.2 Hz	+
Edalatfar et al. [44]	350 ng/√Hz (at 1–5 kHz)	135 dB	4500 Hz	+
Yazdi and Najafi [45]	160 ng/√Hz	--	<1000 Hz	+
Aaltonen et al. [9]	300 ng/√Hz (at 30 Hz)	±1.5 g	300 Hz	+^
Xu et al. [11]	200 ng/√Hz (at 100 Hz)	±1.2 g	700 Hz	^
Utz and Kraft et al. [55]	216 ng/√Hz (at 30–40 Hz)	±1.25 g/5 g	5000 Hz	#-
Kamada and Furubayashi et al. [56]	30 ng/√Hz (at 10–300 Hz)	116 dB	300 Hz	#-^
Krishnamoorthy and Carr et al. [71]	17 ng/√Hz (at 1 Hz)	140 dB	36 Hz	+^&
Williams and Silicon Audio et al. [74]	0.5 ng/√Hz (at 10 Hz)	172 dB	0.005–1.5 kHz	+&
Lu et al. [77]	185.8 ng/√Hz	--	34.5 Hz	+&
Fourguette et al. [87]	10 ng/√Hz	120 dB	80 Hz	+&
Duo et al. [90]	312 ng/√Hz (at 100 Hz)	--	100 Hz	&
Loh et al. [92]	40 ng/√Hz (at 40 Hz)	85 dB (at 40 Hz)	80-1000 Hz	+&
Flores et al. [96]	196 ng/√Hz	--	63,300 Hz	&
Zou and Seshia [100]	144 ng/√Hz (at <1–50 Hz)	±0.05 g	1–50 Hz	+^&
Pandit and Seshia et al. [102]	17.8 ng/√Hz (at 0.25–4 Hz)	±1 g	5 Hz	+^&
Zhao and Seshia et al. [103]	98 ng/√Hz (at 1 Hz)	±1 g	5 Hz	+^&
Yin et al. [104]	380 ng/√Hz	±15 g	2.7 Hz	+^&
Zhao and Seshia et al. [116]	680 ng/√Hz (at 0.5–3 Hz)	--	3 Hz	^&
Abdolvand and Ayazi et al. [62]	213 ng/√Hz (at 2 Hz)	--	200 Hz	+^&
Liu and Kenny [118]	20 ng/√Hz (at 10–1000 Hz)	90 dB	5–1500 Hz	+^&
Liang, Hua and Agafonov et al. [121]	17.8 ng/√Hz (at 1.2 Hz)	--	1.2 Hz	&
Deng et al. [124]	3.2 ng/√Hz (at 0.02 Hz)	±0.01 g	0.2–5 Hz	&
EI Mansouri et al. [21]	17.02 ng/√Hz	--	8.7 Hz	#+^-
Guzman Cervantes et al. [81]	10 ng/√Hz	--	2000 Hz	&
Bao et al. [85]	<1000 ng/√Hz	--	--	&
Zhao et al. [86]	42.4 ng/√Hz	--	29.3 Hz	&
Shin and Kenny et al. [105]	<160 ng/√Hz	--	--	+^&
HP [46].	10 ng/√Hz (at 10–200 Hz)	120 dB	1–200 Hz	+^-
Safran Colibrys SF1500 [48]	300 ng/√Hz (at 10–1000 Hz)	±3 g	0–1500 Hz	+^-
Kinemetrics EpiSensor 2 [49]	3 ng/√Hz (at 1 Hz)	±0.25 g	0–>320 Hz	+-
Reftek Trimble 147A [50]	10 ng/√Hz	±4 g	0–150 Hz	+-
Sercel DSU1-508 [51]	15 ng/√Hz (at 10–200 Hz)	5 g	0–800 Hz	+^-
INOVA ACCUSEIS SL11 [53]	30 ng/√Hz (at 3–400 Hz)	118 dB	3–400 Hz	+-
Honeywell QA3000 [54]	<1000 ng/√Hz	±60 g	>300 Hz	^-+

+: Large proof mass. ^: High Q. #: Low spring constant. -: Low noise interface circuit. &: Novel sensing scheme.

**Table 4:** Summary of micromachined accelerometers with more than µg/√Hz noise floor but a potential to achieve a sub-µg/√Hz noise floor.

Research Group	Noise Floor	Dynamic Range /Full Scale Range	Resonant Frequency	Type
Han et al. [125]	3.0 μg/√Hz (at 0.2–10 Hz)	±0.0029 g	0.2–10 Hz	#
Suzuki et al. [25]	45.0 μg/√Hz	--	37 Hz	#
Krause et al. [94]	10.0 μg/√Hz (at 5–25 kHz)	40 dB	>20,000 Hz	&
